# A C39-like protein from methanogenic archaea: structural insights and functional divergence in β-galactose analogue degradation

**DOI:** 10.3389/fmicb.2026.1895254

**Published:** 2026-09-23

**Authors:** Leizhou Guo, Liuyan Wang, Liping Bai

**Affiliations:** 1 Key Laboratory of Southwest China Wildlife Resources Conservation (Ministry of Education), College of Life Science, China West Normal University, Nanchong, Sichuan, China; 2 Chengdu Institute of Food Inspection, Chengdu, China; 3 Key Laboratory of Development and Application of Rural Renewable Energy, Biogas Institute of Ministry of Agriculture and Rural Affairs, Chengdu, Sichuan, China; 4 Traditional Chinese Medicine Health Industry Promotion Center in Dongxing District, Neijiang, China

**Keywords:** C39-like protein, glycosidase activity, methanogenic archaea, structure and function, β-Galactose analogs

## Abstract

Methanogenic archaea harbor diverse enzymes that support their survival in extreme environments and play key roles in global carbon cycling. The C39 peptidase family, known for cleaving peptide substrates, now gains functional expansion through A0A126R2S0, a novel enzyme that shares structural homology with C39 peptidases (RMSD: 2.0–2.5 Å) but exhibits glycosidase activity. The enzyme harbors a conserved Q-C-H motif in which the third residue of the canonical catalytic triad is substituted from aspartate to asparagine. Enzymatic assays demonstrate that A0A126R2S0 specifically hydrolyzes β-D-galactose analogs, such as p-nitrophenyl-β-D-galactopyranoside (PNPG, Sub 5), with an activity of 16.15 ± 0.71 μM/h, which is 7.8-fold higher than that toward α-configured analogs. Notably, it shows no detectable activity toward double-glycine peptides or archaeal cell wall polypeptides, distinguishing it from canonical C39 peptidases. Truncation experiments confirm that the C-terminal domain alone is sufficient for this glycosidase activity. Molecular docking and site-directed mutagenesis further implicate Loop 3 (residues D314–D324) as a critical determinant of substrate recognition, with the C316-C322 disulfide bond likely stabilizing its conformation. Collectively, these findings expand the functional repertoire of the C39 peptidase superfamily, suggest an evolutionary connection between peptidases and glycosidases, and establish A0A126R2S0 as a candidate biocatalyst for β-galactoside processing.

## 1 Introduction

Methanogenic archaea are essential microorganisms in anaerobic ecosystems, playing a significant role in global carbon cycling, methane emissions, and host-associated processes relevant to human health and disease (Marjory and Hubert, 1933; Adam et al., 2017; Browne et al., 2017; Baker et al., 2020; Borrel et al., 2020; Hoegenauer et al., 2022). Early biochemical studies of these organisms primarily focused on canonical enzymes involved in methanogenesis, particularly methyl-coenzyme M reductase (MCR) (Gendron and Allen, 2022; Müller et al., 2025; Ramírez-Amador et al., 2025), which catalyzes the final step in methane formation. In recent years, however, research has expanded to include newly identified intracellular enzymes involved in methyltransferase activity (Hoeppner et al., 2012; Badmann and Groll, 2020; Kurth et al., 2021; Yu et al., 2023), RNA metabolism (Phung et al., 2017; Dong et al., 2025), environmental signal transduction (Georgiev et al., 2025; Leng et al., 2025; Medina Ferrer and Nayak, 2025), and reverse methanogenic pathways (Scheller et al., 2010; Timmers et al., 2017). Comprehensive investigations of these intracellular enzymes have enhanced our understanding of the adaptive mechanisms of methanogens under extreme physicochemical conditions and their broader implications for biogeochemical carbon cycling, climate feedback systems, and the development of archaeal bioenergy technologies.

C39 peptidases were initially identified as components of bacterial type I ATP-binding cassette (ABC) transport systems. Within these systems, C39 peptidases are localized to the N-terminal domain of the membrane-associated ATPase and typically contain a catalytic triad composed of cysteine, histidine, and aspartate or glutamate residues. Their canonical role involves recognizing leader peptides containing the conserved double glycine motif L(−12)XXXE(−8)L(−7)XXXXG(−2)G(−1) (Havarstein et al., 1994, 1995; Ishii et al., 2010; Lin et al., 2015), followed by cleavage at the double glycine site (GG) during substrate maturation and secretion (Havarstein et al., 1995; Dirix et al., 2004; Nishie et al., 2011; Wu et al., 2012; Kanonenberg et al., 2013). However, recent biochemical analyses have revealed functional diversification within this enzyme family. For instance, the C39 peptidases ThgT from *Thermoanaerobacterium thermosaccharolyticum* and OrgT from *Ornithinibacillus bavariensis* exhibit substrate specificity toward noncanonical Gly-Lys (GK) cleavage sites (Martini et al., 2024), thereby expanding the known substrate range of C39-type proteases. Moreover, a distinct subset of C39 peptidases has been discovered in methanogenic archaea and their associated phages, capable of recognizing isopeptide linkages within the pseudopeptidoglycan layer of archaeal cell walls (Leahy et al., 2010). These enzymes cleave ε-peptide bonds within Glu-γ-Thr/Ala-ε-Lys sequences, underscoring a previously unrecognized functional role of C39 peptidases in archaeal cell envelope modification and phage-host interactions (Altermann et al., 2018; Guo et al., 2026).

During our initial screening of intracellular glycosidases from methanogenic archaea, we identified a previously uncharacterized enzyme, PMBR domain-containing protein A0A126R2S0, which exhibits structural homology to the canonical C39 peptidase scaffold but displays catalytic activity toward p-nitrophenyl (pNP)-modified polysaccharide substrates, which piqued our research interest. This enzymatic behavior diverges from both bacterial C39 peptidases, which target double glycine-containing peptides, and archaeal phage-derived peptidases, which hydrolyze cell wall components, suggesting the emergence of a unique catalytic function. To elucidate the substrate specificity of A0A126R2S0, we systematically screened 10 pNP/oNP-modified aryl glycosides and performed integrated structural and biochemical analyses, including molecular docking, molecular dynamics simulations, and site-directed mutagenesis. The results revealed key residues and structural motifs responsible for substrate recognition and catalysis, suggesting a possible evolutionary connection between C39 peptidases and glycosidases that warrants further investigation. Collectively, this work expands the biochemical repertoire of the C39 peptidase family and provides new insights into enzyme evolution and diversification within methanogenic archaea.

## 2 Materials and methods

### 2.1 Construction of recombinant vectors

Protein sequences A0A126R2S0 and A0A1I4GZX1were retrieved from the UniProt database^1^ (Bateman et al., 2023) and analyzed for signal peptide using SignalP 5.0.^2^

Recombinant plasmids were constructed containing the full-length A0A126R2S0 protein, its N-terminal domain R2S0-ND (residues 1–171), and its catalytic domain R2S0-CD (residues 194–351). Additionally, plasmids were generated containing the catalytic domains GZX1-CD (residues 57–232) of A0A1I4GZX1, 4422-CD (residues 153–32 6) of WP_407454422, 3395-CD (residues 131–308) of WP_400253395, and 6147-CD (residues 199–368) of WP_296796147. The pET28a plasmid was constructed and codon-optimized by Tsingke Biotechnology Co., Ltd. (Beijing, China). For brevity, each protein is referred to by the last four characters of its UniProt ID/Accession Number (e.g., R2S0, 3395) as identifiers. All codon optimization for the selected proteins—including full-length, truncated forms, and catalytic domains—as well as the construction of the pET28a plasmid, was completed by Tsingke Biotechnology Co., Ltd. (Beijing, China).

Mutant plasmids of R2S0-CD were constructed using the Mut Express® II Fast Mutagenesis Kit V2 (C214) from Vazyme Biotechnology Co., Ltd. (Nanjing, China) (see Supplementary Table S1). The required primers, listed in Supplementary Table S2, were synthesized by Sangon Biotech Co., Ltd. (Shanghai, China). All constructed plasmids were then sent to the Chengdu branch of Sangon Biotech Co., Ltd. (Shanghai, China) for sequencing verification.

The protein sequences for the bacterial peptidase C39 domains (LahT147, PCAT1, and ComA) were retrieved from the Protein Data Bank (PDB) under accession codes 6MPZ, 6V9Z, and 3K8U, respectively. The NCBI database was used to obtain the gene sequences of the double-glycine motifs (*LahT147-Sub*, *CtA*, and *ComC*), which correspond to GenBank entries WP_011825069.1, WP_038291094.1, and AJ000870, respectively. All codon optimization and vector construction for the constructs 28a/LahT147, 28a/PCAT1, 28a/ComA, 28a/LahT147-Sub, 28a/CtA, and 28a/ComC were performed by Beijing Tsingke Biotech Co., Ltd. (Beijing, China).

All wild-type and mutant plasmids were initially transformed into *Escherichia coli* DH5α for preservation, followed by transformation into *E. coli* BL21 (DE3) to create expression strains. All mutant proteins were expressed and purified following the same protocol as the wild-type protein (see Section 2.5).

### 2.2 Sequence similarity search

To identify potential homologs of protein A0A126R2S0, we performed a systematic BLASTP search against the NCBI non-redundant (nr) protein sequence database using the BLAST+ suite (version 2.13.0). The query comprised either the full-length or the catalytic domain amino acid sequence of A0A126R2S0. The search employed an E-value threshold of 0.001, a word size of 3, and the BLOSUM62 substitution matrix. To ensure comprehensive coverage, the maximum number of target sequences was set to 500, and filtering for low-complexity regions was disabled to avoid missing distantly related homologs.

### 2.3 Phylogenetic analysis

Phylogenetic analysis was conducted using the Maximum Likelihood (ML) method implemented in MEGA software. The Jones-Taylor-Thornton (JTT) model with Gamma-distributed rate variation among sites (JTT + G) was selected as the substitution model. Gaps and missing data were treated using the partial deletion option with a 95% site coverage cutoff. Branch support was evaluated via Adaptive Bootstrap method. The tree was visualized using the rectangular cladogram layout, and bootstrap values below 50% were not displayed.

### 2.4 Structure superposition and visualization

Structural superimposition was performed using PyMOL (The PyMOL Molecular Graphics System, Version 3.0.3). The two structures were aligned via the super algorithm based on Cα atoms. To visualize the local structural deviations, the ColorByRMSD script (from the PyMOL Wiki) was employed. The resultant root-mean-square deviation (RMSD) values per equivalent residue were mapped onto the 3D structures using a continuous blue-to-red color gradient, where blue indicates minimal deviation and red indicates maximal deviation. Unaligned or flexible loop regions with no equivalent atom pairs were rendered in gray.

### 2.5 Protein expression and purification

The recombinant expression strains were grown in 1 L of LB liquid medium (Yeast Extract 5 g L^−1^, NaCl 10 g L^−1^, Tryptone 10 g L^−1^, pH 7.0) containing 50 μg/mL of kanamycin until OD_600_ reached 0.6 ~ 0.8. IPTG (0.1 mM) was then added, and the cells were incubated at 16 °C for 15 h. Afterward, the cells were collected by centrifugation at 7000 × g. The cells were resuspended in pre-cooled lysis buffer (4 °C, containing 10 mM CaCl_2_, 50 mM Tris, 200 mM NaCl, 1% Triton X-100 (v/v), and 20% glycerol (v/v), pH 7.5) and subjected to ultrasonic disruption on ice (160 W, 5 s on, 8 s off, total 10 min). The disrupted cells were then centrifuged at 15000 × g for 30 min to collect the supernatant for subsequent protein purification. The supernatant was filtered through a 0.45 μm membrane prior to purification. The target protein was purified using a nickel affinity chromatography column (5 mL, HisTrap™HP, Cat# 17524802, Sweden).

The buffer solution for equilibrium consisted of 50 mM Tris, 50 mM imidazole, and 300 mM NaCl at pH 7.5. The elution buffer contained 50 mM Tris, 300 mM imidazole, and 300 mM NaCl at pH 7.5. Imidazole was subsequently removed using a HiPrep™ 26/10 Desalting column (Cat# 17508701, Sweden) with a desalting buffer composed of 20 mM MOPS, 200 mM NaCl, and 10% glycerol (v/v) at pH 7.0. The target protein was then concentrated using an ultrafiltration tube, rapidly frozen in liquid nitrogen, and stored at −80 °C. Protein concentration was quantified using the Bradford method (Bradford, 1976), with bovine serum albumin (BSA) as the standard.

### 2.6 Enzyme activity analysis of pNP/oNP-modified aryl glycosides

p-nitrophenyl-α-D-glucopyranoside (Sub 1), p-nitrophenyl-β-D-glucopyranoside (Sub 2), p-nitrophenyl-N-acetamido-2-deoxy-β-D-glucopyranoside (Sub 3), o-nitrophenyl-β-D-glucopyranoside (Sub 4), p-nitrophenyl-α-D-galactopyranoside (Sub 6) and p-nitrophenyl-α-D-glucofuranoside (Sub 9) were purchased from Shanghai Macklin Biochemical Technology Co., Ltd. (Shanghai, China). p-nitrophenyl-β-D-galactopyranoside (PNPG) (Sub 5) was purchased from Shanghai Aladdin Biochemical Technology Co., Ltd. (Shanghai, China). p-nitrophenyl-N-acetyl-β-D-galactosaminide (Sub 7) was purchased from Sigma Aldrich (Shanghai) Trading Co., Ltd. (Shanghai, China). p-nitrophenyl-β-D-mannopyranoside (Sub 8) was purchased from Shanghai Jizhi Biochemical Technology Co., Ltd. (Shanghai, China). o-nitrophenyl-β-D-galactopyranoside (Sub 10) was purchased from Shenzhen Celmart Technology Co., Ltd. (Shenzhen, China).

The p-nitrophenol or o-nitrophenol-modified polysaccharide substrates were dissolved in 50 mM Bis-Tris propane (pH 7.85) buffer containing 10 mM CaCl₂ and 10 mM TCEP, prepared as a stock solution with a final concentration of 8 mM, and stored at −20 °C. The enzyme activity assay system contained 100 𝜇M pNP/oNP-modified aryl glycosides, with 5 𝜇M enzyme added to initiate the reaction in a total volume of 800 𝜇L using the same buffer. The enzyme-free control was used as the negative reference. All reactions were incubated on a 40 °C shaker, with absorbance readings measured at OD_400_ every 30 min. One enzyme activity unit was defined as the production of 1 𝜇M pNP/oNP per hour at the specified temperature.

### 2.7 Enzyme activity analysis of pNA-modified polypeptides

When EγApNA is the substrate, one enzyme activity unit is defined as the production of 1 μM pNA every 30 min at the specified temperature. For EγSpNA and EγTpNA substrates, one activity unit corresponds to the production of 1 μM pNA every 60 min at the specified temperature. The pNA concentration was determined using a standard curve. The final enzyme concentration was 10 μM, and the final substrate concentration was 100 μM. The enzyme activity assay was conducted in a system containing 50 mM Bis-Tris propane, 10 mM CaCl₂, and 10 mM TCEP at pH 7.85. A buffer solution of the same volume was used in place of the enzyme solution as a negative control to account for non-enzymatic substrate degradation and background signal. The measurement protocol followed the method described by Guo et al. (2025).

### 2.8 Analysis of cleavage activity to double glycine peptide substrates

The enzyme activity reaction system contained a 1:10 molar ratio of enzyme to double glycine peptide, with a total enzyme concentration of 20 μg in a final volume of 20 μL. The enzyme activity buffer consisted of 50 mM Bis-Tris propane, 10 mM CaCl₂, and 10 mM TCEP at pH 7.85. A reaction mixture containing only the double glycine peptide served as the negative control. The reaction was incubated in a 37 °C water bath for 4 h, followed by boiling for 10 min to terminate the reaction. SDS-PAGE electrophoresis was then performed according to the detailed procedures described by Guo et al. (2026).

### 2.9 Enzyme kinetics analysis

Kinetic measurements were carried out using p-nitrophenyl-β-D-galactopyranoside (Sub 5) and o-nitrophenyl-β-D-galactopyranoside (Sub 10). Substrate concentrations ranged from 0.1 to 1.0 mM (0.1, 0.2, 0.3, 0.4, 0.5, 0.8, and 1.0 mM). Each reaction mixture (800 μL) contained 5 μM enzyme in the same buffer used for activity assays. Reactions were started by adding the substrate and incubated at 40 °C on a shaker for 30 min, after which absorbance at 400 nm was recorded. Kinetic parameters (Km and Vmax) were determined by fitting the initial velocity data to the Michaelis–Menten equation (v = Vmax × [S]/(Km + [S])) using nonlinear regression analysis in GraphPad Prism 8.0. The fitting was performed using a least-squares method with equal weighting. Each data point represents the mean of three independent measurements, and the errors for Km and kcat are reported as the standard errors of the fit (SE).

### 2.10 Analysis of the cleavage capacity of lactose

Lactose cleavage was tested in a 100 μL reaction mixture containing 10 μM enzyme and 1.6 mM lactose in 50 mM Bis-Tris propane (pH 7.85). After incubation at 40 °C for 4 h, the reaction was stopped by heating at 95 °C for 15 min in a metal bath. Products were separated by thin-layer chromatography (TLC) on silica gel plates (30 × 100 mm). The mobile phase consisted of n-butanol/ethanol/water (5,3,2 by volume). Samples (3 μL) were spotted three times onto the plate, with drying between applications. Following development, the plate was sprayed with a visualization reagent prepared by mixing solution A (0.1 g diphenylamine in 2.5 mL acetone) and solution B (100 μL aniline in 2.4 mL acetone), then adding 50 μL phosphoric acid and shaking until the white precipitate dissolved. The plate was then heated at 90 °C for 15 min to visualize the spots. D-(+)-galactose and α-D-glucopyranose were used as standards. The protocol was adapted from Wu et al. (2011).

### 2.11 Molecular docking and molecular dynamics simulation

Ligand structures were built using ChemDraw 20.0 and then converted into 3D conformations with Chem3D 20.0 for subsequent energy minimization. Ligands were preprocessed using the molecular docking software MOE (version 2022.02) to generate appropriate conformations and isomers. The processed ligands were saved in the ligand database in the “.mdb” format. Receptor proteins were further refined using the QuickPrep module in MOE, and a blind cavity search was initially performed using the Site Finder module to detect potential ligand-binding pockets across the entire protein surface based on geometric and energetic criteria. This analysis identified a top-ranked concave cleft within the canonical V-shaped groove of the C39-like domain as the putative active-site pocket. Subsequently, this pocket was defined as the docking grid center for site-specific docking. Molecular docking was performed using MOE with an induced-fit protocol that allowed selected side chains of the binding pocket to be flexible. The software was configured to generate minimum energy complexes and 20 different conformations. The predicted binding pocket was visually inspected to ensure it corresponded to the active-site cleft. The top three poses were selected by the GBVI/WSA dG scoring function, which estimates the binding free energy. The results were ranked according to the calculated binding energy, the S-score, using the London dG function for initial localization and the GBVI/WSA dG function for optimized localization. Finally, the molecular interactions of the predicted ligand-protein complexes were analyzed using PyMol.

The top three optimal conformations obtained by molecular docking were used as the starting structures for MD simulation using GROMACS (version 2024.2) (Van Der Spoel et al., 2005). Each protein-ligand complex was solvated in a dodecahedral box with the TIP3P water model, and the system was neutralized by adding Na^+^ or Cl^−^ ions to a final concentration of 0.15 M NaCl. Energy minimization was performed using the steepest descent method with a maximum force tolerance of 100 kJ·mol^−1^·nm^−1^. The system was then equilibrated under NVT (constant number of particles, volume, and temperature) conditions at 300 K for 100 ps, followed by NPT (constant number of particles, pressure, and temperature) equilibration at 1 atm for 100 ps. Production runs were conducted at 300 K and 1 atm for 100 ns with a 2 fs time step, with coordinates saved every 10 ps for subsequent analysis, using the CHARMM36m force field (Huang et al., 2017).

### 2.12 Statistical analysis

We performed all statistical tests with GraphPad Prism 8.0. Experimental results represent the means ± SD from three replicates. To determine significance, we used one-way ANOVA followed by Dunnett’s *post hoc* test for multiple group comparisons, and paired *t*-tests where applicable. Significance levels are defined as *p < 0.05, **p < 0.01, ***p < 0.001, and ****p < 0.0001, while “ns” indicates *p* ≥ 0.05.

## 3 Results

### 3.1 A0A126R2S0 contains a C39-like domain

Due to the inability to obtain crystal diffraction data for A0A126R2S0, we predicted its structure using the AlphaFold Service^3^ (Abramson et al., 2024). The protein comprises two domains: the N-terminal pseudopeptidoglycan-binding domain (PB) and the C-terminal catalytic domain (CD), with a predicted pTM score of 0.59 (Figure 1A). Despite the moderate global pTM score for the full-length predicted structure (pTM > 0.5 indicates the overall fold is likely correct), the catalytic domain of A0A126R2S0 (R2S0-CD, residues 194–351) yielded a pTM value of 0.9, indicating high confidence in the global fold of this domain. Consistently, per-residue pLDDT analysis showed that the catalytic domain is predominantly modeled with high confidence (Supplementary Figure S1A). Furthermore, the full-chain PAE matrix showed that positional errors for most residue pairs within the catalytic domain fell within 5 Å, suggesting global structural stability (Supplementary Figures S1A,B). In parallel, neither the SMART database^4^ nor the Conserved Domain Database (CDD) of NCBI^5^ annotated the catalytic domain of A0A126R2S0 as belonging to any known functional domain (Figure 1B).

**Figure 1:**
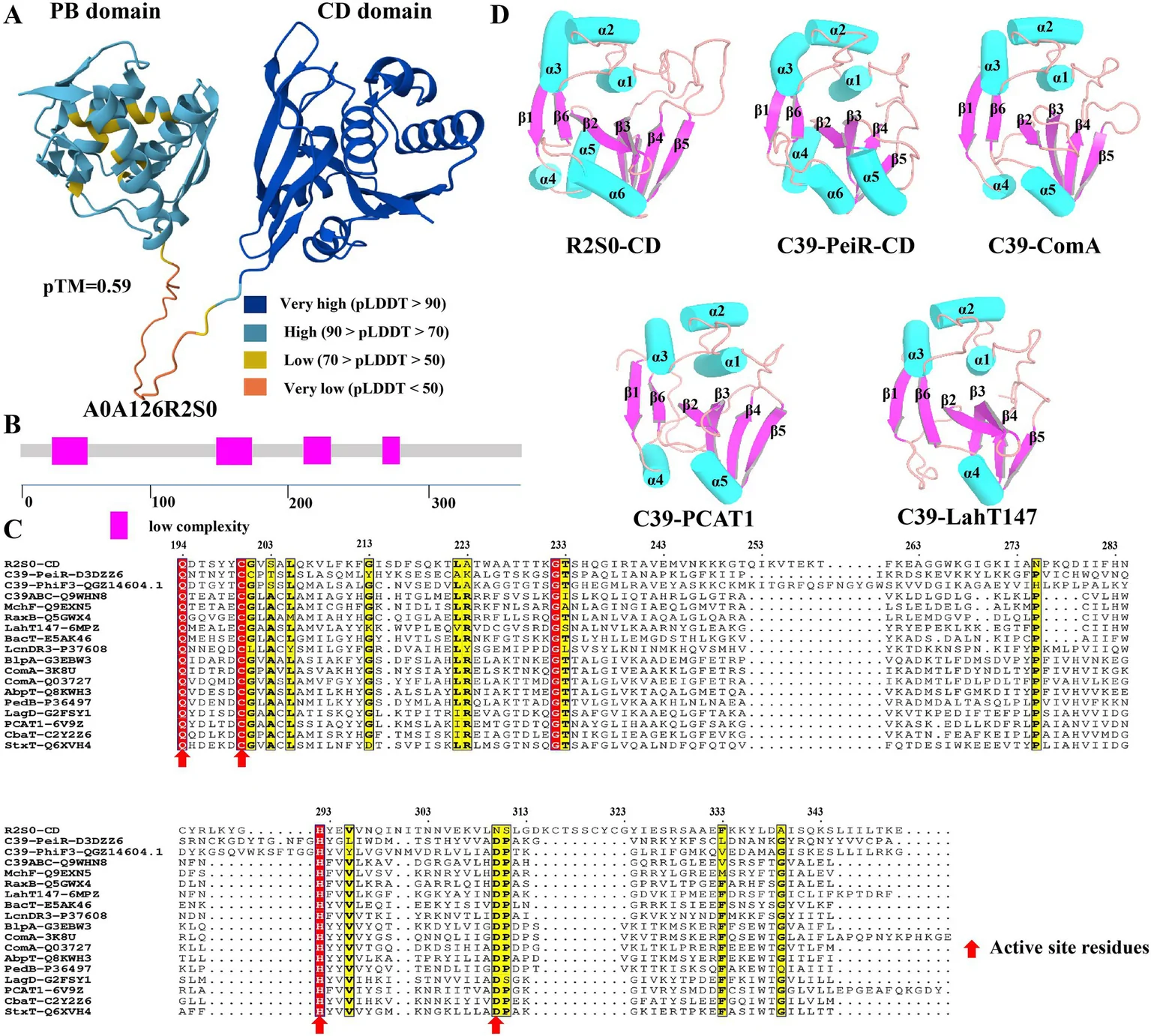
Structural prediction of A0A126R2S0. **(A)** Structure of A0A126R2S0 predicted by AlphaFold 3, colored by pLDDT (predicted Local Distance Difference Test), a per-residue confidence score where blue indicates high confidence and red indicates low confidence. **(B)** Conserved domain prediction of A0A126R2S0 using the SMART online prediction server. **(C)** Schematic representation of C39 peptidases from different sources; secondary structures are numbered, with alpha helices depicted as cyan cylinders. **(D)** Sequence similarity analysis between A0A126R2S0 and C39 peptidases.

To explore the potential function of R2S0-CD, we used its protein sequence to query the NCBI online BLAST server (BlastSearch&LINK_LOC = blasthome)^6^ under the parameters detailed in section 2.7 of Materials and Methods. The search returned 144 homologous sequences, 120 of which were derived from methanogenic archaea (Supplementary Table S3). A breakdown of their functional annotations showed that, among these 144 proteins, 79 were annotated as hypothetical proteins, 24 as C39 family peptidases or C39 domain-containing proteins, 19 as peptidoglycan-binding proteins, 8 as Ig-like domain repeat proteins, and 7 as putative glycoside hydrolases. This distribution pointed to a relatively high degree of homology between R2S0-CD and C39 peptidases. A closer inspection of the sequence alignment, however, revealed that R2S0-CD shares with bacterial C39 peptidases only the cysteine and histidine residues of the conserved catalytic triad; the third residue, which is typically an aspartate, is replaced by an asparagine (Figure 1C).

To further classify its classification, several C39 peptidases with known structures and well-characterized functions were selected for analysis. The catalytic domain R2S0-CD of A0A126R2S0 exhibits low sequence identity with the catalytic domains of C39-PeiR (PDB code: 8Z4H) (Guo et al., 2026), C39-ComA (PDB code: 3K8U) (Ishii et al., 2010), C39-PCAT1 (PDB code: 6V9Z) (Kieuvongngam et al., 2019), and C39-LahT147 (PDB code: 6MPZ) (Bobeica et al., 2019), with sequence identities of 17.26, 17.68, 15.24, and 11.59%, respectively. It is generally accepted that proteins with sequence identities below 20–25% are likely from different families or represent distant homologs. From a sequence perspective, direct inheritance of identical sites from a common ancestor is rare. Collectively, these observations argue that R2S0-CD does not belong to the C39 peptidase family.

Given the low sequence identity between R2S0-CD and canonical C39 peptidases, it was unexpected to find a high degree of structural similarity. A structural homology search via the Foldseek server^7^ revealed that the R2S0-CD domain showed E-values of 1.17 × 10^−6^ and 1.12 × 10^−5^ for the C39 peptidases Q8DQW1 and P59653 (PDB ID: 8HF4) from *Streptococcus pneumoniae* R6, respectively, and an E-value of 2.24 × 10^−5^ for the C39 peptidase Q0PC75-F1 from *Campylobacter jejuni*. Furthermore, the structural superposition of R2S0-CD with the catalytic domains of C39-PeiR-CD, C39-ComA, C39-PCAT1, and C39-LahT147 gave RMSD values of 2.0, 2.5, 2.5, and 2.4 Å, respectively (Supplementary Figure S2A). All these structures contain six central β-sheets flanked by four to six α-helices on both sides (Figure 2C and Supplementary Figure S2B). The marked conservation of this core topology and the arrangement of secondary structural elements accentuate a remarkable phenomenon: proteins with highly divergent sequences can adopt nearly identical folds.

**Figure 2:**
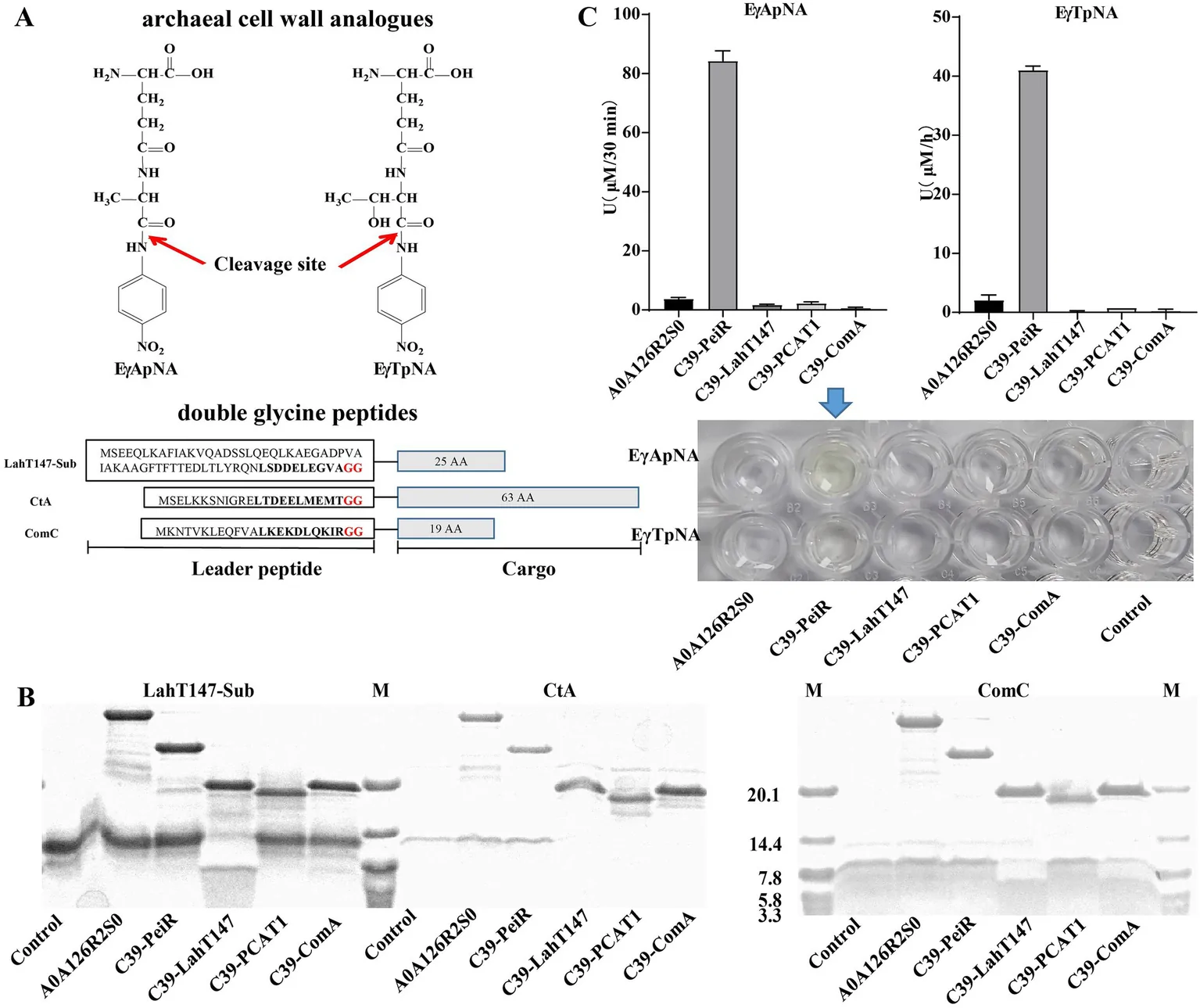
Comparison of the cleavage capabilities of A0A126R2S0 toward C39 peptidase substrates from various sources. **(A)** Structural schematic diagram of archaeal cell wall analogs EγApNA and EγTpNA. The arrow indicates the cleavage site **(B)** Analysis of the cleavage activity of A0A126R2S0 toward bacterial double glycine peptide substrates LahT147-Sub, CtA, and ComC **(C)** Analysis of the cleavage activity of A0A126R2S0 toward methanogenic archaeal cell wall analogs EγApNA and EγTpNA.

Taken together, these structural and sequence comparisons lead us to classify A0A126R2S0 as a C39-like protein—one that exhibits low sequence identity but high structural resemblance to canonical C39 peptidases—and its catalytic domain is hence designated as the C39-like domain.

### 3.2 A0A126R2S0 exhibits distinct functional characteristics compared to the reported C39 peptidases

The high structural similarity between A0A126R2S0 and C39 peptidases raises the possibility that they might share analogous biological roles. The reported biological functions of C39 peptidases are as follows: (1) the ability of C39 peptidases derived from methanogenic archaeal phages to cleave archaeal cell walls (e.g., C39-PeiR) (Altermann et al., 2018; Guo et al., 2026); and (2) the double glycine-cleaving ability of bacterial C39 peptidases (e.g., C39-ComA, C39-PCAT1, and C39-LahT147).

To test whether A0A126R2S0 possesses either of these endopeptidase activities, we heterologously expressed and purified the aforementioned enzymes together with their cognate double-glycine peptide substrates. For the archaeal cell-wall assays, the native isopeptide substrates were replaced with para-nitroanilide (pNA)-modified peptide analogues (Figure 2A). The cleavage results of bacterial-derived double glycine peptide substrates showed that LahT147, PCAT1, and ComA were fully capable of cleaving CtA. LahT147 and ComA were also able to cleave ComC, but only LahT147 could cleave LahT147-sub (Figure 2B). This finding is consistent with previous results (Guo et al., 2026). In contrast, similar to C39-PeiR, A0A126R2S0 showed no cleavage activity against the three double glycine polypeptide substrates: LahT147-Sub, CtA, and ComC (Figure 2B). Conversely, when the substrates were cell wall analogs from methanogenic archaea, only C39-PeiR demonstrated significant cleavage activity against EγApNA and EγTpNA (Figure 2C). In contrast, A0A126R2S0 exhibited negligible cleavage activity against pNA-modified cell wall peptides, comparable to LahT147, PCAT1, and ComA (Figure 2C). Taken together, our data suggest that A0A126R2S0 represents a previously unrecognized member of the C39-like superfamily that has lost the canonical endopeptidase activities, and its true biological substrate remains to be identified.

### 3.3 A0A126R2S0 can cleave β-galactose analogs

UniProt annotation^8^ identifies A0A126R2S0 as a PMBR domain-containing protein. The PMBR (pseudopeptidoglycan-binding repeat) is a cell-wall-binding motif predominantly found in archaeal cell-wall lytic enzymes. Given that A0A126R2S0 exhibited virtually no cleavage of the isopeptide analogues EγApNA and EγTpNA, we next sought to determine whether it might hydrolyze the glycan chains of the pseudopeptidoglycan—the other major component of the archaeal cell envelope. Because native archaeal cell walls are structurally complex and difficult to obtain as purified, homogeneous glycan polymers, we instead purchased nine commercially available polysaccharide substrates modified with either para-nitrophenol (pNP) or ortho-nitrophenol (oNP) (Figure 3A).

**Figure 3:**
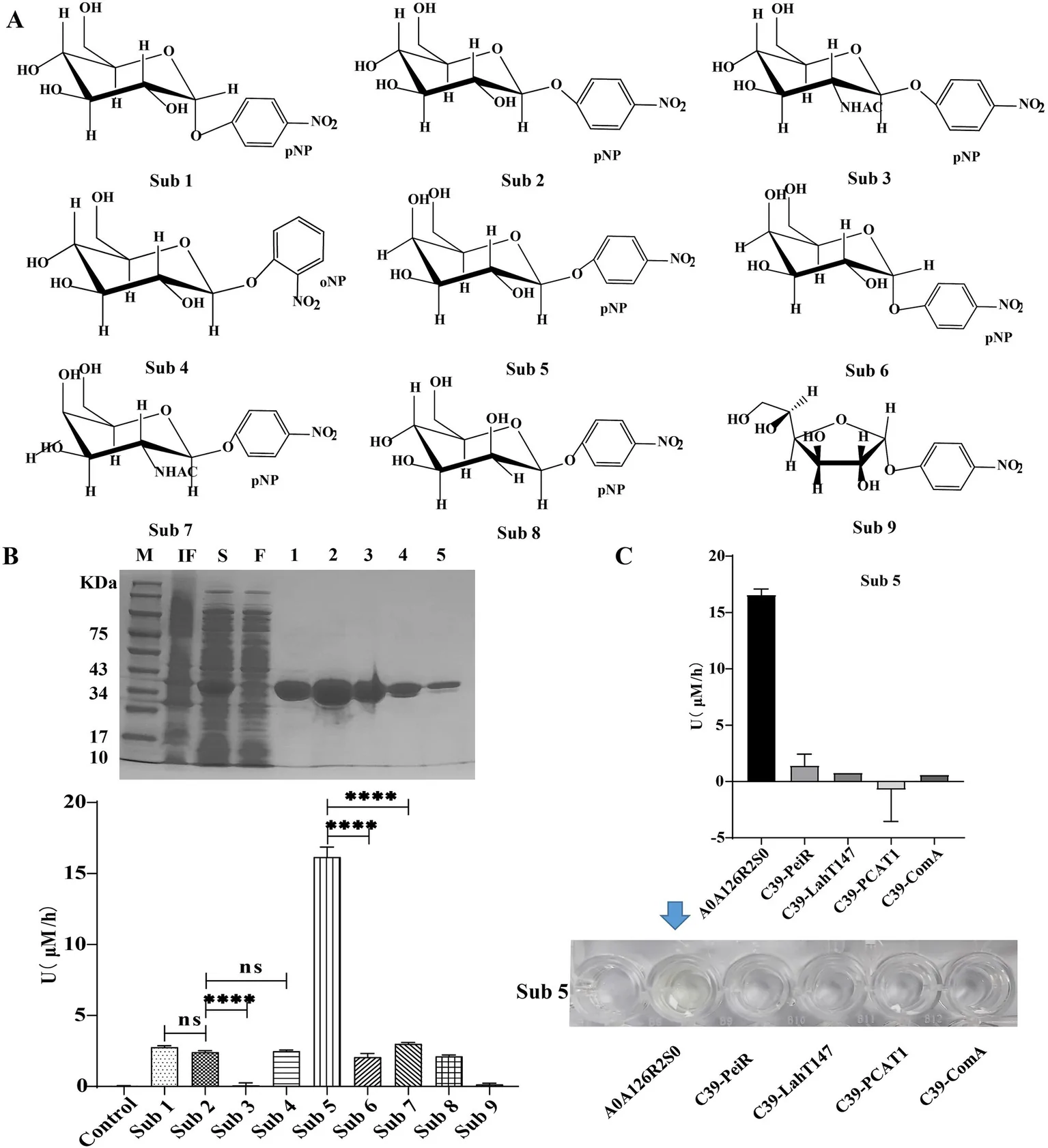
Comparison of the cleavage capabilities of A0A126R2S0 toward pNP- and oNP-modified aryl glycosides. **(A)** Schematic representation of the structures of pNP- and oNP-modified aryl glycosides. **(B)** Comparison of the relative cleavage activities of A0A126R2S0 toward pNP- and oNP-modified aryl glycosides. SDS-PAGE analysis of the purified A0A126R2S0 protein. Note: M, PageRuler Plus Prestained Protein Ladder; IF, insoluble fraction; S, supernatant; F, flow-through. **(C)** Analysis of the cleavage activity of C39 peptidases from various sources toward p-nitrophenyl-β-D-galactopyranoside (Sub 5).

Substrates 1 and 2, linked at position 4 of pNP, are D-glucopyranose in the α and β configurations, respectively. Substrate 3 differs from substrate 2 in that the hydroxyl group at position 2 of D-glucopyranose is replaced by an N-acetyl amino group. The difference between substrate 4 and substrate 2 is that β-D-glucopyranose is linked at position 2 of oNP. Substrates 5 and 6 are linked to D-galactopyranose in the β and α configurations, respectively, at position 4 of pNP. Substrate 7 is derived from substrate 5 by the addition of an N-acetyl amino group at position 2 of β-D-galactopyranose. Substrate 8 contains β-configuration D-pyranomannan at position 4 of pNP, and substrate 9 contains α-D-furanoglucose at position 4 of pNP.

Enzyme activity results showed that A0A126R2S0 exhibited weak cleavage ability toward pNP- or oNP-modified glucopyranose, and neither the configuration of glucopyranose nor the position of the nitro group on the modified benzene ring significantly affected enzyme activity (Figure 3B, Substrates 1, 2, and 4). However, when the hydroxyl group at position 2 of glucopyranose was replaced by a larger N-acetyl group (Substrate 3), A0A126R2S0 lost its substrate cleavage capability. For galactose analogues, the cleavage ability of A0A126R2S0 toward D-galactopyranose in the β-configuration was 7.8 times greater than that in the α-configuration, with enzyme activity reaching 16.15 ± 0.71 μM/h. When the hydroxyl group at position 2 of β-D-galactopyranose was replaced by a larger N-acetylamino group, enzyme activity significantly decreased, indicating that A0A126R2S0 has substrate specificity for β-D-galactopyranose, as the addition of any group may lead to loss of enzyme activity (Figure 3B, Substrates 5, 6, and 7). Additionally, A0A126R2S0 showed weak cleavage ability toward β-D-pyranomannan (Substrate 8), but almost no cleavage ability toward the polysaccharide modification of α-D-glucofuranose (Substrate 9) (Figure 3B). As controls, we also tested C39-PeiR, LahT147, PCAT1, and ComA under identical conditions. None of these enzymes showed appreciable activity against p-nitrophenyl-β-D-galactopyranoside (substrate 5) (Figure 3C).

Collectively, these glycan hydrolysis data further underscore the functional divergence of A0A126R2S0 from canonical C39 peptidases: while it lacks the expected isopeptide and double-glycine cleavage activities, it displays a selective, albeit modest, glycosidase activity toward specific β-galactoside derivatives, hinting at a distinct biological role that remains to be defined.

### 3.4 The catalytic domain of A0A126R2S0 exerts cleavage capacity

To evaluate the correlation or independence between the cell wall binding domain and the catalytic domain of A0A126R2S0 in the cleavage of pNP-modified aryl glycosides, we constructed and purified two truncated proteins: R2S0-ND, containing only the N-terminal PB domain (residues 1–171), and R2S0-CD, containing only the C-terminal CD domain (residues 172–351) (Figures 4A,B).

**Figure 4:**
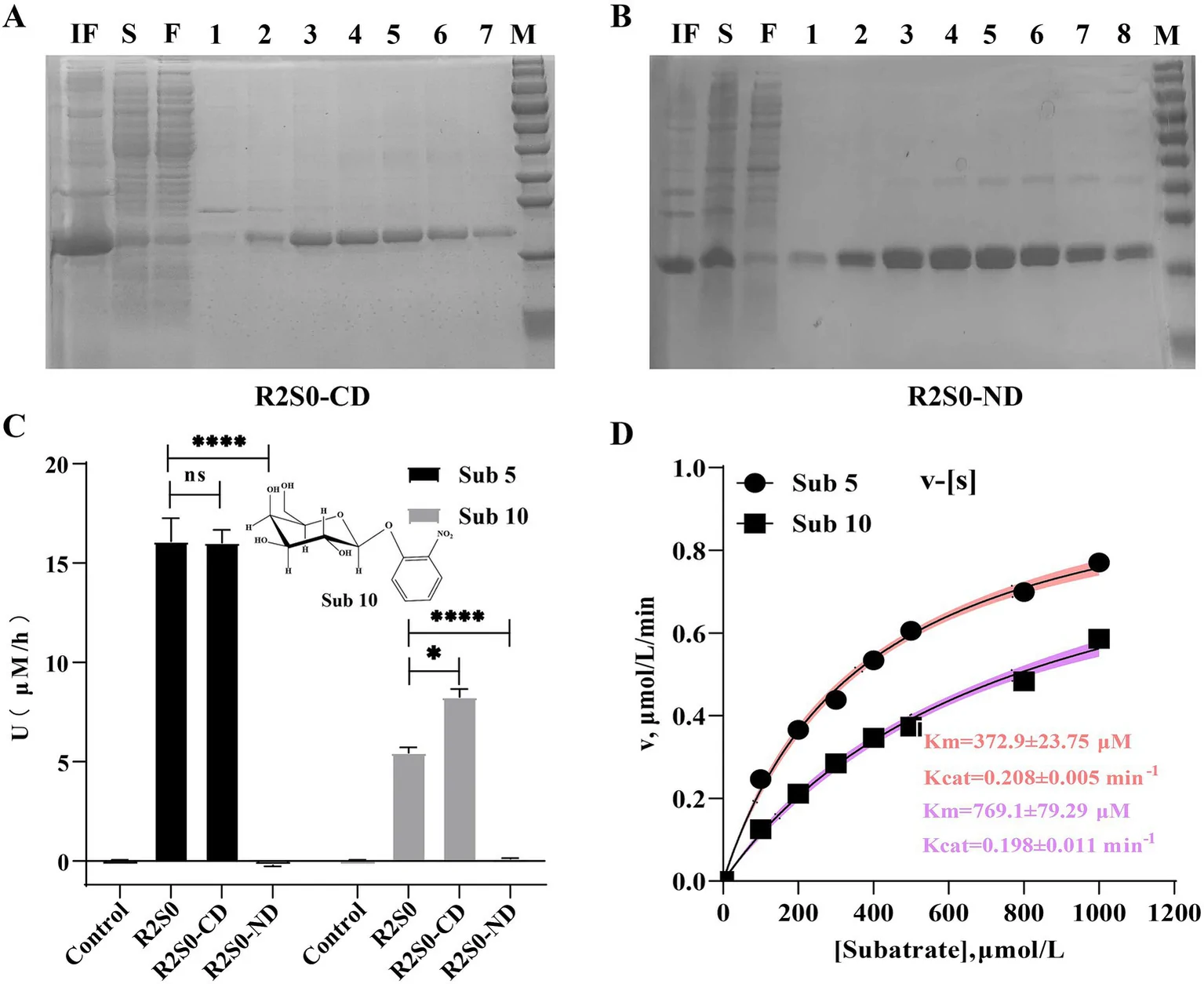
Functional analysis of truncated variants of A0A126R2S0. **(A)** Purification results of the catalytic domain protein R2S0-CD from A0A126R2S0. **(B)** Purification results of the N-terminal cell wall-binding domain R2S0-ND from A0A126R2S0. **(C)** Analysis of the cleavage activity of A0A126R2S0 truncated variants toward p-nitrophenyl-β-D-galactopyranoside (Substrate 5) and 2-nitrophenyl-β-D-galactopyranoside (Substrate 10). **(D)** Enzyme kinetic analysis of R2S0-CD and R2S0-ND.

Enzymatic activity assays demonstrated that the truncated R2S0-CD exhibited cleavage activity comparable to that of the full-length protein A0A126R2S0 against p-nitrophenyl-β-D-galactopyranoside (PNPG) (Substrate 5), whereas R2S0-ND showed no cleavage activity, consistent with the control group (Figure 4C). When the substrate was replaced with o-nitrophenyl-β-D-galactopyranoside (Substrate 10), similar results were observed. These findings indicate that the C-terminal catalytic domain of A0A126R2S0 is independently responsible for polysaccharide cleavage.

Additionally, we characterized the enzymatic kinetics of protein R2S0-CD using two substrates: p-nitrophenyl-β-D-galactopyranoside (Sub 5) and o-nitrophenyl-β-D-galactopyranoside (Sub 10). The Km values for these substrates were 372.9 ± 23.75 μM and 769.1 ± 79.29 μM, respectively, with catalytic constants (kcat) of 0.208 ± 0.005 min^−1^ and 0.198 ± 0.011 min^−1^ (Figure 4D). The calculated catalytic efficiencies (kcat/Km) for Sub 5 and Sub 10 were (5.58 ± 0.38) × 10^−4^ and (2.57 ± 0.28) × 10^−1^ mM^−1^·min^−1^, respectively, indicating that R2S0-CD exhibits approximately 2.2-fold higher catalytic efficiency toward the para-nitrophenyl substrate (Sub 5) than toward the ortho-nitrophenyl substrate (Sub 10) (Supplementary Table S5).

### 3.5 Loop 3 of A0A126R2S0 is a critical region for modified polysaccharide cleavage ability

Given the absence of co-crystal structures of R2S0-CD with its substrates, we performed site-specific molecular docking using MOE software to explore potential binding modes between the catalytic domain and the 10 glycan derivatives. For each substrate, 20 docking poses were generated, and the three top-scoring conformations were retained for further analysis. Their calculated binding free energies and interacting residues are summarized in Supplementary Table S4.

A survey of the entire docking dataset revealed that C200 (present in substrates 1, 2, 3, 4, 5, 6, 9, and 10) and G291 (found in substrates 1, 2, 3, 4, 5, 6, 7, and 10) are the two most frequently occurring residues. This pattern suggests that all substrates share a common, rudimentary binding pocket composed of residues such as C200 and G291, which may provide non-specific initial anchoring forces. Notably, nearly every substrate displayed distinct combinations of interacting residues across its three top conformations, implying that the binding pocket is intrinsically flexible and can accommodate multiple substrate orientations.

Although substrates 3 and 7 showed marginally better docking scores, their binding involved only weakly polar or hydrophobic residues (e.g., G291, H292, and T230). Substrate 8, despite a relatively low calculated energy (−6.0285 kcal/mol), lacked any polar residue anchoring—a case of “high-energy, low-quality” binding. While the MOE docking scores did not rank substrate 5 as the overall strongest binder (the energy difference from the best-scoring substrate being less than 0.6 kcal/mol, well within the computational error range), it exhibited a remarkably distinctive binding mode. Substrate 5 was the only compound whose most favorable conformations simultaneously engaged both S197 and Y321 in polar interactions. This unique polar anchoring pattern distinguishes it from other high-affinity substrates that relied primarily on hydrophobic contacts involving residues such as G291 or H292.

For R2S0-CD with substrate 5, the three top docking conformations yielded scores of −5.6, −5.5, and −5.5 kcal/mol. In all three binding modes, substrate 5 was positioned within the characteristic concave cleft of the C39-like domain. In pose 1, the p-nitrophenyl ring of p-nitrophenyl-β-D-galactopyranoside (substrate 5) was oriented toward the left side of the active-site pocket, whereas in poses 2 and 3, the same ring was directed to the right side (Figure 5A).

**Figure 5:**
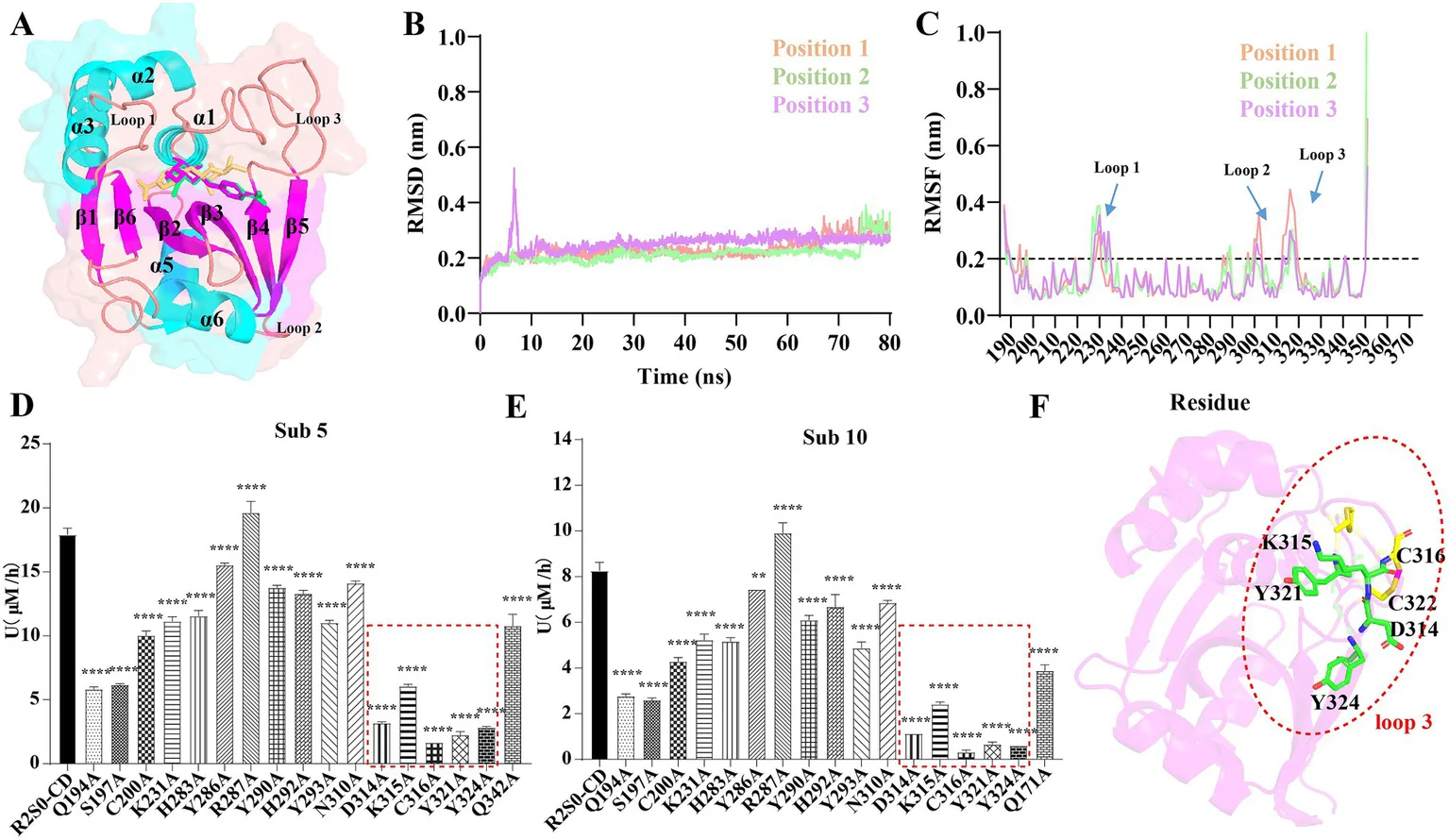
Site-specific molecular docking and molecular dynamics simulation results of R2S0-CD with p-nitrophenyl-β-D-galactopyranoside (Substrate 5). **(A)** Three binding conformations of the substrate p-nitrophenyl-β-D-galactopyranoside (Sub 5) within the V-shaped binding pocket of R2S0-CD. **(B, C)** RMSD and RMSF analyses of the docking results shown in **(A)**. **(D)** Analysis of the cleavage capability of R2S0-CD mutant proteins toward Substrate 5. **(E)** Analysis of the cleavage capability of R2S0-CD mutant proteins toward Substrate 10. **(F)** Illustration of key residues influencing the enzymatic activity of R2S0-CD.

To further identify potential residues involved in substrate recognition during the binding process, we carried out molecular dynamics simulations on the three optimal docking conformations of R2S0-CD with substrate 5. Throughout the simulation trajectories, the RMSD values of the three binding modes within this region remained relatively stable, without any pronounced fluctuations. Meanwhile, analysis of the RMSF profiles revealed that residues in loops 1, 2, and 3 underwent substantial conformational rearrangements during the docking process (Figures 5B,C).

Guided by the molecular docking and dynamics simulations, we next performed site-directed mutagenesis to validate the putative recognition residues within the “V”-shaped pocket. All mutant proteins showed migration patterns consistent with the wild-type on SDS-PAGE, confirming that the introduced point mutations did not grossly affect protein folding or stability. Among the 17 mutants generated, substitutions at Q194, S197, and K315 reduced the enzymatic activity to approximately 30% of the wild-type level, whereas mutations at D314, C316, Y321, and Y324 led to even more pronounced losses in activity (Figures 5D,E). Notably, five of these critical residues—D314, K315, C316, Y321, and Y324—are all located within loop 3, highlighting the essential contribution of this region to substrate recognition (Figure 5F). Furthermore, the marked activity reduction observed for the S197A and Y321A mutants aligns well with our docking predictions, in which both residues were identified as key polar anchors for substrate 5. This consistency strongly validates the reliability of the predicted binding mode between R2S0-CD and its preferred glycan substrate.

## 4 Discussion

### 4.1 Identification and substrate specificity of A0A126R2S0

In this study, a previously uncharacterized enzyme, A0A126R2S0, was identified from a methanogenic archaeon and shown to exhibit novel glycosidase activity toward β-D-galactose analogs. Structural predictions and comparisons revealed that the overall fold of this enzyme closely resembles that of the C39 peptidase family. Although the sequence identity between A0A126R2S0 and typical C39 peptidases (e.g., C39-PeiR, C39-ComA, C39-PCAT1, and C39-LahT147) is below 20%, significant structural similarity suggests they may share a common ancestral fold and some biological functions. Notably, A0A126R2S0 harbors a conserved catalytic dyad (Cys-His), yet the third residue of the canonical triad is substituted by asparagine instead of the typically conserved aspartate, implying that its catalytic mechanism may diverge from that of conventional C39 peptidases.

Enzymatic assays further illuminated the functional specificity of A0A126R2S0. The enzyme displayed no detectable cleavage activity toward double-glycine peptide substrates (LahT147-Sub, CtA, and ComC) or archaeal cell-wall isopeptide analogues (EγApNA and EγTpNA). In contrast, it exhibited pronounced substrate selectivity toward the p-nitrophenyl-modified β-D-galactoside (substrate 5). Truncation experiments demonstrated that the C-terminal catalytic domain (R2S0-CD) alone is sufficient to accomplish substrate hydrolysis, suggesting that this region functions as a self-contained catalytic unit. Molecular docking revealed a distinctive binding mode for substrate 5 within the R2S0-CD pocket: it was the only compound among all tested substrates that simultaneously engaged both S197 and Y321 in polar interactions in its most favorable conformations. Site-directed mutagenesis verified the critical roles of these two residues, as the S197A and Y321A mutants both exhibited markedly reduced activity, supporting their involvement in substrate anchoring or catalytic activation. Moreover, mutations at D314, K315, C316, and Y324—all located within loop 3—led to even more severe losses of activity. Coupled with the substantial conformational fluctuations observed in this region during molecular dynamics simulations, these results underscore the essential contribution of loop 3 to substrate recognition.

Taken together, the multilayered evidence from structural prediction, enzymatic characterization, molecular simulations, and mutagenesis supports the view that A0A126R2S0 represents a functionally diverged member of the C39 peptidase superfamily. Its substrate preference has shifted from typical peptide or cell-wall components toward glycosidic substrates, exemplifying a case of functional repurposing during enzyme evolution. Given its marked sequence divergence from canonical C39 peptidases and its functional departure from conventional peptidase activity, classifying A0A126R2S0 as a typical C39 peptidase would be inappropriate. We therefore propose that this enzyme and its homologues be assigned to a distinct group—the C39-like protein family—to distinguish them from the functionally conserved C39 peptidases.

### 4.2 Potential of A0A126R2S0 toward natural substrates

Pseudopeptidoglycan is a distinctive cell wall component of methanogenic archaea, characterized by a glycan backbone composed of N-acetylglucosamine and N-acetyl talosaminuronic acid linked by β-1,4 glycosidic bonds. The N-terminus of A0A126R2S0 contains a putative pseudopeptidoglycan-binding (PB) domain, a motif previously shown to target pseudopeptidoglycan in other proteins (Pfister et al., 1998; Steenbakkers et al., 2006; Visweswaran et al., 2011b, 2011a). However, the presence of this domain alone does not imply cell wall-lytic activity; its actual function in A0A126R2S0 requires experimental validation. Moreover, direct verification of its activity toward natural glycan chains is currently impeded by the structural complexity and limited accessibility of native pseudopeptidoglycan oligosaccharides—neither well-defined, homogeneous preparations are commercially available, nor is a scalable synthetic route yet established to supply these substrates at the purity and quantity required for reliable enzymatic assays.

Despite these experimental constraints, the substrate profiling with pNP-modified aryl glycosides offered indirect but informative clues. A0A126R2S0 was unable to hydrolyze the pNP-modified polysaccharide containing N-acetylglucosamine (Sub 3) but specifically cleaved p-nitrophenyl-β-D-galactopyranoside (PNPG, Sub 5). This selectivity suggests that the enzyme may have limited capacity to cleave the β-1,4-glycosidic linkage between N-acetylD-glucosamine and N-acetyl-L-talosaminuronic acid units, and its natural target might not be the pseudopeptidoglycan backbone per se. It should be noted, however, that pNP-modified glycans cannot fully recapitulate the intricate architecture of the native cell wall, and thus these inferences warrant cautious interpretation.

To further characterize its substrate specificity, the enzyme’s ability to hydrolyze the natural disaccharide lactose (β-D-galactopyranose linked to α-D-glucopyranose via a β-1,4 bond) was evaluated. Neither the full-length A0A126R2S0 protein nor its isolated catalytic domain was able to cleave lactose (Supplementary Figure S4). This result does not contradict the activity observed with pNP-modified β-D-galactopyranoside; a likely explanation is that lactose adopts a more complex three-dimensional structure and exists as rotamers in aqueous solution, which increases steric hindrance and impedes binding to the active site.

Despite the inability to cleave lactose, the enzyme’s preference for β-D-galactopyranose remains informative. Galactose is also a component of the extracellular cell wall of methanogenic archaea, where it serves various physiological functions and is particularly abundant in the genus *Methanobacterium* (Kandler and Konig, 1993). Given that the full-length protein contains a PB domain, it is worth investigating whether A0A126R2S0 can cleave galactose moieties in the cell wall and thereby influence physiological processes such as extracellular signaling, cell-wall remodeling, or growth regulation. Future studies are warranted to perform enzymatic assays with native methanogenic archaeal cell-wall oligosaccharides and to elucidate the physiological relevance of this enzyme in its natural host.

### 4.3 Functional divergence, evolutionary implications, and conservation/diversity of loop 3

Although the catalytic domain of A0A126R2S0 shares the fundamental C39 peptidase scaffold with C39-PeiR-CD, C39-ComA, C39-PCAT1, and C39-LahT147, as evidenced by low RMSD values indicating high structural similarity, their functions differ significantly. Detailed structural analysis revealed several key distinctions. For example, alignment with C39-PeiR-CD showed that C39-PeiR contains an additional α-helix 7 outside the core C39 scaffold, which is absent in A0A126R2S0. Conversely, A0A126R2S0 features a longer loop 3 region on the right side of its active pocket that is not present in C39-PeiR (Supplementary Figure S5A). Nevertheless, some residues are conserved, such as the “C-H” residues forming the catalytic dyad (C90 and H181 in C39-PeiR, corresponding to C200 and H292 in A0A126R2S0), the oxyanion hole–forming residue “Q” (Q84 in C39-PeiR, Q194 in A0A126R2S0), and residues H162 and Y182, which are critical for substrate recognition in C39-PeiR (corresponding to H283 and Y293 in A0A126R2S0) (Supplementary Figure S5A). Notably, mutations of these key residues only reduced, but did not abolish, the enzymatic activity of A0A126R2S0, indicating that the enzyme does not strictly depend on them for polysaccharide cleavage.

Similarly, C39-ComA, C39-PCAT1, and C39-LahT147 lack the loop 3 region on the right side of the active pocket, which may explain why these C39 peptidases are unable to cleave polysaccharide analogs (Figure 2B). Conversely, the presence of this loop 3 region in A0A126R2S0 might obstruct its active pocket from accommodating bulkier double glycine peptide substrates. These findings reinforce that the glycan-cleaving activity of R2S0-CD is distinct from that of archaeal cell wall lytic enzymes and bacterial double glycine endopeptidases. Furthermore, previously characterized C39 peptidases require high concentrations of calcium ions to cleave archaeal cell walls or bacterial double glycine peptides. In contrast, R2S0-CD can cleave polysaccharide analogs under physiological conditions (i.e., without additional Ca^2+^) (Supplementary Figure S5B). This Ca^2+^-independent polysaccharide cleavage activity represents another important distinction from C39 peptidases such as C39-PeiR.

These structural and functional divergence described above has significant evolutionary implications. This analysis is consistent with the hypothesis of an evolutionary connection between the C39 peptidase family and glycosidases, although this hypothesis requires further validation with expanded phylogenetic sampling and structural studies. Despite low sequence similarity (<20% identity), the high structural similarity and conserved catalytic core suggest a shared ancient ancestral fold. Divergence in substrate specificity likely resulted from gene duplication events followed by adaptive mutations in substrate-binding regions, particularly in loop 3. The disulfide bond that stabilizes the conformation of loop 3 further underscores the importance of this region in substrate positioning and recognition.

To assess the conservation and functional relevance of loop 3, we constructed a phylogenetic tree comprising 159 proteins, including 15 bacterial C39 peptidases and 144 homologues of R2S0-CD retrieved from Supplementary Table S3 (Supplementary Figure S6). In this tree, the bacterial C39 peptidases formed a discrete clade (branch 4) consisting solely of the basic C39 scaffold. Proteins in branch 1 all contained a conserved loop 3 region; branch 2 contained both loop 3 and the α7-helix–loop characteristic of C39-PeiR; and branch 3 harbored only the α7-helix–loop. Four representative homologs were selected for heterologous expression (Supplementary Figure S6B, marked with triangles); However, only 3395-CD was obtained as a soluble protein (Supplementary Figure S7A). Activity assays revealed that 3395-CD exhibited minimal ability to cleave polysaccharide analogs but showed pronounced cleavage activity toward pNA-modified archaeal cell wall peptides, with relative activities of 118.02 ± 24.04 U for EγApNA and 48.84 ± 1.44 U for EγTpNA (Supplementary Figures S7B,C). Additionally, it was capable of hydrolyzing bacterial double glycine peptides (LahT147-Sub and CtA) (Supplementary Figure S7D). To our knowledge, this is the first report of the C39-like protein. Although 3395-CD shares 40.85% sequence identity with R2S0-CD and both contain a conserved loop 3, their enzymatic activities differ markedly.

Sequence alignment revealed that residues critical for R2S0-CD activity (e.g., S197, K231, Y290, D314, K315) are not conserved in 3395-CD (Supplementary Figure S8). The C316–C322 disulfide bond observed in R2S0-CD likely stabilizes the conformation of loop 3, suggesting that the primary role of this loop may be structural—maintaining the architecture of the active pocket—whereas the fine specificity toward β-galactoside substrates is determined by non-conserved residues such as S197, D314, K315, and Y321. This interpretation is consistent with the observation that 3395-CD, despite sharing loop 3, displays a completely different substrate profile, as the corresponding specificity-determining residues are not conserved between the two proteins. However, we acknowledge that pNA-modified substrates do not fully replicate native cell wall structures, and the precise relationship between loop 3 conservation and substrate specificity warrants further investigation.

### 4.4 Analysis of the polysaccharide cleavage mechanism and biochemical significance of A0A126R2S0

Based on catalytic mechanisms, glycoside hydrolases are generally classified into inverting and retaining enzymes (Koshland, 1953), the main difference being whether the anomeric configuration of the cleavage product is inverted relative to the substrate. Both mechanisms rely on two key amino acid residues (glutamate or aspartate), typically separated by 6–11 Å (McCarter and Stephen Withers, 1994). Analysis of residues near the loop 3 region in the active pocket of R2S0-CD identified three potential acidic residues that might participate in the cleavage reaction: D314, E326, and E332. The distance between OD2 of D314 and OE2 of E326 is 13.9 Å, while the distance between OE2 of E326 and OE2 of E332 is 5.1 Å (Supplementary Figure S9A). The importance of D314 was previously validated (Figure 5 D and E). In this study, the functions of E326 and E332 were further examined. Mutation of E326 or E332 did not significantly reduce the cleavage activity of R2S0-CD toward Sub 5; instead, the E332 mutation slightly increased the activity (Supplementary Figure S9B). These results argue against a direct catalytic role for either of these two acidic residues in the glycan-hydrolyzing activity of R2S0-CD.

In addition to the two classical glycoside hydrolase mechanisms, several distinct mechanisms have been reported in recent years, including: (1) glycosyl-phosphate lyases that lack a general acid, where the general acid (usually glutamate) is replaced by glutamine (Tiels et al., 2012); (2) a mechanism involving neighboring-group participation, in which the N-acetyl group at the C-2 position of certain substrates acts as an intramolecular nucleophile to form an oxazolinium ion intermediate (Terwisscha Van Scheltinga et al., 1995; Sobala et al., 2020); (3) some retaining glycoside hydrolases (e.g., plant myrosinases) can utilize substrate functional groups or exogenous bases to stabilize the transition state when hydrolyzing glucosinolates, thereby reducing dependence on a general acid (Burmeister et al., 2000); (4) families GH33 and GH34 employ tyrosine as the nucleophilic residue (Watts et al., 2003; Amaya et al., 2004). In addition, NAD-dependent mechanisms, exemplified by the GH4 family, proceed through redox reactions coupled with elimination/addition steps without traversing a classical oxocarbenium-ion transition state (Rajan et al., 2004; Yip et al., 2004). Notably, the Y321 mutation substantially impaired the activity of R2S0-CD in our assays, hinting at a potential role for this residue in substrate positioning or catalysis. However, in the absence of crystal structures of A0A126R2S0 or R2S0-CD—either alone or in complex with substrates—we cannot definitively assign the catalytic mechanism at this stage.

Importantly, while these observations implicate several residues in the catalytic process, the precise roles of D314, E326, E332, and Y321—and whether R2S0-CD follows an inverting, retaining, or non-canonical mechanism—remain speculative in the absence of direct chemical or structural evidence, such as kinetic isotope effects, product stereochemistry analysis, or co-crystal structures.

The discovery of a glycosidase within a molecular framework resembling C39 peptidases underscores the remarkable biochemical diversity of this enzyme superfamily. The enzyme’s ability to specifically cleave β-D-galactose analogs, rather than peptides or archaeal cell wall components, suggests it may play a specialized role in archaeal carbohydrate metabolism. This function could be crucial for adaptation to extreme environments or for energy acquisition. The enzyme’s moderate catalytic efficiency and substrate specificity indicate potential biotechnological applications, such as serving as a biocatalyst for the modification or degradation of β-galactose-containing aryl glycosides. Future elucidation of its catalytic mechanism would enhance our understanding of archaeal enzymatic strategies, a field that remains underexplored compared to bacterial systems.

### 4.5 Limitations and future directions

Although this study provides strong evidence for the enzyme’s activity and structural basis, several key questions require further experimental validation. First, determining the three-dimensional structure of the enzyme—ideally in complex with a substrate analog—using X-ray crystallography or cryo-electron microscopy would clarify its substrate-binding mode and catalytic mechanism. Second, expanding the substrate range to include native polysaccharides directly extracted from archaea will help elucidate the enzyme’s physiological function. Third, investigating homologous proteins from other archaeal species or environmental samples may reveal the prevalence and diversity of such glycosidases across the archaeal domain. Finally, exploring the regulatory mechanisms and expression patterns of this enzyme under natural environmental conditions will help uncover its biological role in archaeal ecosystems.

## 5 Conclusion

In summary, the data presented here support the conclusion that A0A126R2S0 represents a novel glycosidase whose structural framework resembles that of canonical C39 peptidases, yet whose function has diverged substantially. This finding reveals an unexpected evolutionary trajectory within an enzyme superfamily and highlights the remarkable capacity for functional diversification. These insights not only advance our understanding of enzyme evolution but also open new possibilities for biotechnological applications. Future studies focused on structural validation and ecological function will further elucidate the significance of this enzyme in archaeal biology and biogeochemical cycles.

## Data Availability

The data analyzed in this study were obtained from publicly available repositories, including UniProt, NCBI, and PDB. AlphaFold-predicted structural models for A0A126R2S0 generated in this study are provided in the Supplementary material. No new experimental datasets were generated or deposited in external repositories. All other data are included in the article/Supplementary material.

## Footnotes

1
https://www.uniprot.org/uniprotkb

2
https://services.healthtech.dtu.dk/services/SignalP-5.0/

3
https://alphafoldserver.com/

4
https://smart.embl.de/

5
https://www.ncbi.nlm.nih.gov/cdd

6
https://blast.ncbi.nlm.nih.gov/Blast.cgi?PROGRAM=blastp&PAGE_TYPE=

7
https://search.foldseek.com/search

8
https://www.uniprot.org/uniprotkb
