# Supplementary material for: A C39-like protein from methanogenic archaea: structural insights and functional divergence in β-galactose analogue degradation
Source: Front Microbiol. 2026 Sep 23;17:1895254. doi: 10.3389/fmicb.2026.1895254 (PMC13642728; doi:10.3389/fmicb.2026.1895254)
Supplement: Supplementary file 1 [file Table_1.DOCX]

Supplementary Materials for

A C39-Like Protein from Methanogenic Archaea: Structural Insights and Functional Divergence in β‑Galactose Analogue Degradation

Leizhou Guo1,2,3,*, Liuyan Wang4, Liping Bai1,*

1Key Laboratory of Southwest China Wildlife Resources Conservation (ministry of education), College of Life Science, China West Normal University, Nanchong, Sichuan 637009, China

2Chengdu Institute of Food Inspection, Chengdu 611130, China

3Key Laboratory of Development and Application of Rural Renewable Energy, Biogas Institute of Ministry of Agriculture and Rural Affairs, Chengdu, Sichuan 610041, China

4Traditional Chinese Medicine Health Industry Promotion Center in Dongxing District, Neijiang, 641100, China

* Correspondence:

Corresponding Author

Leizhou Guo, [guoleizhou@caas.cn](mailto:guoleizhou@caas.cn) and Liping Bai., [bailiping039@163.com](mailto:bailiping039@163.com)

**This PDF file includes:**

Figures S1 to S9

Tables S1 to S5

**Figure S1. AlphaFold-predicted structure and confidence assessment of R2S0-CD.**

(A) Predicted three-dimensional structure of R2S0-CD. The protein backbone is color-coded according to the per-residue predicted Local Distance Difference Test (pLDDT) scores, with numerical confidence values (ranging from 0 to 100) indicated alongside each residue position. Dark blue regions (pLDDT > 90) indicate very high confidence in the local backbone geometry, while light blue/green regions (90 > pLDDT > 70) denote high confidence. Yellow regions (70 > pLDDT > 50) represent low confidence, and orange/red regions (pLDDT < 50) indicate very low confidence, typically suggesting intrinsically disordered or flexible segments. (B) Predicted Aligned Error (PAE) plot. This two‑dimensional matrix displays the expected positional error (in Ångströms, scale 0-30 Å) of residue x when the structural model is aligned on residue y, where both axes correspond to the residue numbers of R2S0-CD. Low PAE values (dark-colored areas) indicate that the relative spatial positions between the corresponding residue pairs (or distinct domains) are predicted with high confidence. Conversely, high PAE values (light-colored areas) indicate substantial uncertainty in their relative orientation, and thus these inter-domain or inter-residue distances should not be interpreted as reliable. The prominent dark diagonal line (representing self-alignment with zero error) is biologically meaningless and should be disregarded during interpretation.

**Figure S2. Superimposed structures of R2S0-CD and C39 peptidases derived from various sources.**

(A) Cartoon representations of superimposed structures are colored according to the local structural deviations (per-residue RMSD) derived from pairwise Cα alignments. The color gradient ranges from blue (indicating minimal local displacement, i.e., high structural conservation) to red (indicating maximal local displacement, i.e., pronounced conformational variability). Regions that could not be unambiguously aligned, predominantly flexible loop segments, are rendered in gray. This coloring scheme highlights the conserved structural cores versus the variable surface regions among the compared proteins. (B) Topological comparison of domain architectures between R2S0-CD and C39 family homologs. Protein identities are labeled on the left or adjacent to each bar. This comparative topological representation enables a direct visual assessment of the conservation and divergence in domain numbers, linear order, and inter-domain linker lengths between R2S0-CD and its homologs, thereby providing structural insights into their potential functional specialization and evolutionary relationships.

**Figure S3. Molecular docking results of R2S0-CD with substrate 5 and substrate 6.**

(A) Optimal binding pose of R2S0-CD with substrate 5: The ligand is shown as green sticks , and key interacting residues are displayed as thin sticks with labels (residue name and number). Potential hydrogen bonds are indicated as yellow dashed lines. (B) Optimal binding pose of R2S0-CD with substrate 6: The representation is identical to that in (A), with substrate 6 colored in the same scheme.

**Figure S4.** **Lactose cleavage activity of A0A126R2S0 and its truncated protein R2S0-CD.**

**Figure S5. Comparative analysis of the active pockets of R2S0-CD and C39-PeiR.**

(A) Conserved residues in the active pockets of R2S0-CD and C39-PeiR. The red dashed box indicates the loop 3 region unique to R2S0-CD, and the green dashed box indicates the α-helix 7 unique to C39-PeiR. (B) Ca²⁺ concentration dependence of the cleavage activity of R2S0-CD. "+" indicates addition of extra Ca²⁺ to the enzyme reaction system, and "–" indicates no extra Ca²⁺ added.

**Figure S6. Homology analysis of the loop 3 region in R2S0-CD.**

**Figure S7. Substrate specificity analysis of the truncated protein 3395-CD.**

(A) SDS‑PAGE analysis of purified 3395‑CD. (B) Cleavage activity of 3395‑CD against ten pNP/oNP‑modified polysaccharides. (C) Cleavage activity of 3395‑CD against the archaeal cell wall analogs EγApNA and EγTpNA. (D) Cleavage activity of 3395‑CD against the bacterial‑derived double glycine peptide substrates LahT147‑Sub, CtA, and ComC.

**Figure S8. Sequence alignment of R2S0-CD and WP_400253395.**

**Figure S9. Functional validation of key residues in the active pocket of R2S0-CD.**

(1) Relative spatial distribution of D314, E326, and E332 in the active pocket. (2) Enzymatic activity analysis of the E326 and E332 mutants.

**Table S1 Strains and plasmids used in this study**

| **Plasmids** | **Description** | **Source** |
| --- | --- | --- |
| **Plasmids** |  |  |
| pET28a（+） | *Kanr oripBR322 laclq T7p* | Novagen |
| 28a/A0A126R2S0 | pET28a-derived plasmid carrying the *A0A126R2S0* gene | Tsingke Biotech |
| 28a/R2S0-ND | pET28a-derived plasmid carrying the *R2S0-ND* gene | Tsingke Biotech |
| 28a/R2S0-CD | pET28a-derived plasmid carrying the *R2S0-CD* gene | Tsingke Biotech |
| 28a/R2S0-CDΔQ23A | pET28a-derived plasmid carrying the *R2S0-CDΔQ23A* gene | This study |
| 28a/R2S0-CDΔS26A | pET28a-derived plasmid carrying the *R2S0-CDΔS26A* gene | This study |
| 28a/R2S0-CDΔC29A | pET28a-derived plasmid carrying the *R2S0-CDΔC29A* gene | This study |
| 28a/R2S0-CDΔK60A | pET28a-derived plasmid carrying the *R2S0-CDΔK60A* gene | This study |
| 28a/R2S0-CDΔH112A | pET28a-derived plasmid carrying the *R2S0-CDΔH112A* gene | This study |
| 28a/R2S0-CDΔY115A | pET28a-derived plasmid carrying the *R2S0-CDΔY115A* gene | This study |
| 28a/R2S0-CDΔR116A | pET28a-derived plasmid carrying the *R2S0-CDΔR116A* gene | This study |
| 28a/R2S0-CDΔY119A | pET28a-derived plasmid carrying the *R2S0-CDΔY119A* gene | This study |
| 28a/R2S0-CDΔH121A | pET28a-derived plasmid carrying the *R2S0-CDΔH121A* gene | This study |
| 28a/R2S0-CDΔY122A | pET28a-derived plasmid carrying the *R2S0-CDΔY122A* gene | This study |
| 28a/R2S0-CDΔN139A | pET28a-derived plasmid carrying the *R2S0-CDΔN139A* gene | This study |
| 28a/R2S0-CDΔD143A | pET28a-derived plasmid carrying the *R2S0-CDΔD143A* gene | This study |
| 28a/R2S0-CDΔK144A | pET28a-derived plasmid carrying the *R2S0-CDΔK144A* gene | This study |
| 28a/R2S0-CDΔC145A | pET28a-derived plasmid carrying the *R2S0-CDΔC145A* gene | This study |
| 28a/R2S0-CDΔY150A | pET28a-derived plasmid carrying the *R2S0-CDΔY150A* gene | This study |
| 28a/R2S0-CDΔY153A | pET28a-derived plasmid carrying the *R2S0-CDΔY153A* gene | This study |
| 28a/R2S0-CDΔQ171A | pET28a-derived plasmid carrying the *R2S0-CDΔQ171A* gene | This study |
| 28a/LahT147-Sub | pET28a-derived plasmid carrying the *LahT147-Sub* gene | Tsingke Biotech |
| 28a/CtA | pET28a-derived plasmid carrying the *CtA* gene | Tsingke Biotech |
| 28a/ComC | pET28a-derived plasmid carrying the *ComC* gene | Tsingke Biotech |
| 28a/LahT147 | pET28a-derived plasmid carrying the *LahT147* gene | Tsingke Biotech |
| 28a/PCAT1 | pET28a-derived plasmid carrying the *PCAT1* gene | Tsingke Biotech |
| 28a/ComA | pET28a-derived plasmid carrying the *ComA* gene | Tsingke Biotech |
| 28a/LahT147-Sub | pET28a-derived plasmid carrying the *LahT147-Sub* gene | Tsingke Biotech |
| 28a/3395-CD | pET28a-derived plasmid carrying the *3395-CD* gene | Tsingke Biotech |
| *E. coli* BL21(DE3) | F^-^ *omp*T hsdSB(rB^-^ mB^-^ ) *gal dcm*(DE3) | Sangon Biotech |
| *E. coli* DH5α | F^-^ *end*A1 *gln*V44 *thi*-1 *rec*A1 *rel*A1 *gyr*A96 *deo*R *nup*G φ 80d*lac*ZΔM15 Δ(*lac*ZYA-*arg*F)U169, *hsd*R17(rK^-^ mK^+^ ),λ^-^ | Sangon Biotech |

**Table S2 List of primers required for construction of recombinant *E. coli* strain**

| **Gene name** | **Primer name** | **Sequence（5’-3’）** | **Fragment size** |
| --- | --- | --- | --- |
| *R2S0-CDΔQ23A* | EP1 | TATGGGT**gcg**GACACCAGCTACTACTGCGGTG | 5781 bp |
|  | EP2 | TGGTGTC**cgc**ACCCATACCACCACAACCCTGA |  |
| *R2S0-CDΔS26A* | EP3 | AGACACC**gcg**TACTACTGCGGTGTTTCTGCTCTG | 5781 bp |
|  | EP4 | AGTAGTA**cgc**GGTGTCTTGACCCATACCACCA |  |
| *R2S0-CDΔC29A* | EP5 | CTACTAC**gcg**GGTGTTTCTGCTCTGCAGAAAGT | 5781 bp |
|  | EP6 | AAACACC**cgc**GTAGTAGCTGGTGTCTTGACCCATAC |  |
| *R2S0-CDΔK60A* | EP7 | AACCACCACC**gcg**GGTACCTCTCACCAGGGTATCCG | 5781 bp |
|  | EP8 | TACC**cgc**GGTGGTGGTTGCTGCCCAAGTCGCC |  |
| *R2S0-CDΔH112A* | EP9 | CATCTTC**gcg**AACTGCTACCGTCTGAAATACGG | 5781 bp |
|  | EP10 | AGCAGTT**cgc**GAAGATGATGTCTTGTTTCGGATTC |  |
| *R2S0-CDΔY115A* | EP11 | TGC**gcg**CGTCTGAAATACGGTCACTACGAAGT | 5781 bp |
|  | EP12 | TATTTCAGACG**cgc**GCAGTTGTGGAAGATGATGTCTTG |  |
| *R2S0-CDΔR116A* | EP13 | CTGCTAC**gcg**CTGAAATACGGTCACTACGAAGTTG | 5781 bp |
|  | EP14 | ATTTCAG**cgc**GTAGCAGTTGTGGAAGATGATGTCTT |  |
| *R2S0-CDΔY119A* | EP15 | TCTGAAA**gcg**GGTCACTACGAAGTTGTTAACCAGAT | 5781 bp |
|  | EP16 | AGTGACC**cgc**TTTCAGACGGTAGCAGTTGTGG |  |
| *R2S0-CDΔH121A* | EP17 | ATACGGT**gcg**TACGAAGTTGTTAACCAGATCAACATC | 5781 bp |
|  | EP18 | CTTCGTA**cgc**ACCGTATTTCAGACGGTAGCAGT |  |
| *R2S0-CDΔY122A* | EP19 | GTCAC**gcg**GAAGTTGTTAACCAGATCAACATCACC | 5781 bp |
|  | EP20 | AACAACTTC**cgc**GTGACCGTATTTCAGACGGTAGC |  |
| *R2S0-CDΔN139A* | EP21 | AAAGTGCTG**gcg**TCTCTGGGTGACAAATGCACC | 5781 bp |
|  | EP22 | AGAGA**cgc**CAGCACTTTCTCAACGTTGTTGGT |  |
| *R2S0-CDΔD143A* | EP23 | TCTGGGT**gcg**AAATGCACCTCTAGCTGCTATTGC | 5781 bp |
|  | EP24 | TGCATTT**cgc**ACCCAGAGAGTTCAGCACTTTCTC |  |
| *R2S0-CDΔK144A* | EP25 | TGAC**gcg**TGCACCTCTAGCTGCTATTGCGGTT | 5781 bp |
|  | EP26 | TAGAGGTGCA**cgc**GTCACCCAGAGAGTTCAGCACTT |  |
| *R2S0-CDΔC145A* | EP27 | TGACAAA**gcg**ACCTCTAGCTGCTATTGCGGTT | 5781 bp |
|  | EP28 | TAGAGGT**cgc**TTTGTCACCCAGAGAGTTCAGCA |  |
| *R2S0-CDΔY150A* | EP29 | TGC**gcg**TGCGGTTACATCGAGTCTCGTTCTGC | 5781 bp |
|  | EP30 | ATGTAACCGCA**cgc**GCAGCTAGAGGTGCATTTGTCA |  |
| *R2S0-CDΔY153A* | EP31 | TATTGCGGT**gcg**ATCGAGTCTCGTTCTGCAGCG | 5781 bp |
|  | EP32 | TCGAT**cgc**ACCGCAATAGCAGCTAGAGGTGCA |  |
| *R2S0-CDΔQ171A* | EP33 | GATCTCT**gcg**AAATCTCTGATCATTCTGACCAAAGA | 5781 bp |
|  | EP34 | GAGATTT**cgc**AGAGATCGCGTCCAGGTACTTC |  |

Note: The black lowercase codon is the mutation site.

**Table S3 Homologous protein sequences of R2S0-CD.**

| **Description** | **Scientific Name** | **Max Score** | **Total Score** | **Query Cover** | **E value** | **Per. ident** | **Acc. Len** | **Accession** |
| --- | --- | --- | --- | --- | --- | --- | --- | --- |
| hypothetical protein [Methanobrevibacter olleyae] | *Methanobrevibacter olleyae* | 327 | 327 | 100% | 7e-110 | 100 | 351 | WP_067148754.1 |
| hypothetical protein [uncultured Methanobrevibacter sp.] | *uncultured Methanobrevibacter sp.* | 274 | 274 | 99% | 9e-89 | 80.89 | 348 | WP_295618857.1 |
| hypothetical protein [Methanobrevibacter olleyae] | *Methanobrevibacter olleyae* | 261 | 261 | 100% | 3e-85 | 81.65 | 232 | WP_074798179.1 |
| hypothetical protein [Methanobrevibacter sp.] | *Methanobrevibacter sp.* | 258 | 258 | 99% | 2e-82 | 82.69 | 352 | WP_406533417.1 |
| hypothetical protein, partial [Methanobrevibacter sp.] | *Methanobrevibacter sp.* | 216 | 216 | 84% | 3e-66 | 81.2 | 322 | WP_458454603.1 |
| hypothetical protein [Methanobrevibacter sp.] | *Methanobrevibacter sp.* | 207 | 207 | 80% | 2e-65 | 76.98 | 128 | MBO7443224.1 |
| hypothetical protein [Methanobrevibacter sp.] | *Methanobrevibacter sp.* | 186 | 186 | 99% | 2e-54 | 60.51 | 329 | MBQ2652659.1 |
| pseudomurein-binding repeat-containing protein [uncultured Methanobrevibacter sp.] | *uncultured Methanobrevibacter sp.* | 181 | 181 | 99% | 9e-53 | 59.87 | 329 | WP_296874596.1 |
| cysteine peptidase family C39 domain-containing protein [Methanobrevibacter sp.] | *Methanobrevibacter sp.* | 177 | 177 | 100% | 6e-51 | 56.96 | 329 | WP_439779166.1 |
| peptidoglycan-binding protein [Methanosphaera sp.] | *Methanosphaera sp.* | 165 | 165 | 100% | 4e-46 | 52.94 | 350 | WP_455644820.1 |
| MAG: hypothetical protein BZ134_00335 [Methanosphaera sp. SHI1033] | *Methanosphaera sp. SHI1033* | 160 | 160 | 91% | 2e-44 | 53.69 | 353 | RAP45805.1 |
| pseudomurein-binding repeat-containing protein [Methanobrevibacter sp.] | *Methanobrevibacter sp.* | 156 | 156 | 99% | 3e-43 | 50.96 | 305 | WP_458454412.1 |
| hypothetical protein HMCMC_IBMIKG_01736 [uncultured Methanobrevibacter sp.] | *uncultured Methanobrevibacter sp.* | 151 | 151 | 100% | 7e-43 | 49.37 | 180 | CAQ6822295.1 |
| hypothetical protein HMCMC_MMBDGF_00787 [uncultured Methanobrevibacter sp.] | *uncultured Methanobrevibacter sp.* | 151 | 151 | 100% | 1e-42 | 48.73 | 180 | CAQ8776486.1 |
| hypothetical protein HMCMC_LJPJEP_00831 [uncultured Methanobrevibacter sp.] | *uncultured Methanobrevibacter sp.* | 154 | 154 | 100% | 3e-42 | 50 | 308 | CAQ7370047.1 |
| hypothetical protein HMCMC_MLNDIO_00350 [Methanobrevibacter oralis] | *Methanobrevibacter oralis* | 150 | 150 | 100% | 3e-42 | 49.37 | 180 | CAR3682260.1 |
| hypothetical protein [Methanobrevibacter sp. V74] | *Methanobrevibacter sp. V74* | 154 | 154 | 100% | 3e-42 | 50 | 314 | WP_305555241.1 |
| hypothetical protein [Methanobrevibacter sp.] | *Methanobrevibacter sp.* | 153 | 153 | 100% | 8e-42 | 46.2 | 328 | WP_458456071.1 |
| hypothetical protein HMCMC_DMMHIF_01144 [uncultured Methanobrevibacter sp.] | *uncultured Methanobrevibacter sp.* | 152 | 152 | 100% | 9e-42 | 49.37 | 314 | CAQ8338781.1 |
| hypothetical protein HMCMC_ADLGGK_00046 [uncultured Methanobrevibacter sp.] | *uncultured Methanobrevibacter sp.* | 151 | 151 | 100% | 4e-41 | 48.73 | 314 | CAR2805687.1 |
| hypothetical protein HMCMC_NBCPFO_00944 [uncultured Methanobrevibacter sp.] | *uncultured Methanobrevibacter sp.* | 150 | 150 | 100% | 5e-41 | 48.73 | 314 | CAQ7458281.1 |
| hypothetical protein HMCMC_CNCJDG_01119 [uncultured Methanobrevibacter sp.] | *uncultured Methanobrevibacter sp.* | 150 | 150 | 100% | 5e-41 | 48.73 | 314 | CAQ7447186.1 |
| hypothetical protein HMCMC_DKFOIC_00369 [Methanobrevibacter oralis] | *Methanobrevibacter oralis* | 150 | 150 | 100% | 6e-41 | 49.37 | 314 | CAR2664958.1 |
| hypothetical protein HMCMC_KJKDLF_00342 [uncultured Methanobrevibacter sp.] | *uncultured Methanobrevibacter sp.* | 150 | 150 | 100% | 6e-41 | 49.37 | 314 | CAQ6898219.1 |
| hypothetical protein HMCMC_AGEOMI_01795 [uncultured Methanobrevibacter sp.] | *uncultured Methanobrevibacter sp.* | 150 | 150 | 100% | 7e-41 | 48.73 | 314 | CAQ8116483.1 |
| hypothetical protein HMCMC_LFFCAK_00074 [Methanobrevibacter oralis] | *Methanobrevibacter oralis* | 150 | 150 | 100% | 1e-40 | 49.37 | 314 | CAQ7896184.1 |
| hypothetical protein [Methanobrevibacter sp.] | *Methanobrevibacter sp.* | 150 | 150 | 99% | 1e-40 | 47.13 | 326 | WP_407454422.1 |
| hypothetical protein [Methanobrevibacter sp.] | *Methanobrevibacter sp.* | 148 | 148 | 99% | 1e-39 | 46.15 | 336 | MBP3226406.1 |
| peptidoglycan-binding protein [Methanosphaera cuniculi] | *Methanosphaera cuniculi* | 143 | 143 | 100% | 1e-37 | 47.88 | 353 | WP_170104103.1 |
| hypothetical protein [Bacilli bacterium] | *Bacilli bacterium* | 147 | 147 | 100% | 5e-37 | 46.2 | 757 | MBR3208642.1 |
| hypothetical protein [Bacteroidales bacterium] | *Bacteroidales bacterium* | 140 | 140 | 100% | 5e-37 | 44.3 | 313 | MEE1227208.1 |
| putative glycoside hydrolase [Methanobrevibacter sp.] | *Methanobrevibacter sp.* | 142 | 142 | 100% | 7e-36 | 45.57 | 588 | WP_292749837.1 |
| pseudomurein-binding repeat-containing protein [Methanobrevibacter sp.] | *Methanobrevibacter sp.* | 135 | 135 | 100% | 5e-35 | 43.67 | 321 | WP_405282128.1 |
| cysteine peptidase family C39 domain-containing protein [Methanobrevibacter sp.] | *Methanobrevibacter sp.* | 134 | 134 | 99% | 9e-35 | 45.06 | 297 | WP_458454203.1 |
| glycoside hydrolase family protein [uncultured Methanobrevibacter sp.] | *uncultured Methanobrevibacter sp.* | 137 | 137 | 99% | 6e-34 | 44.94 | 586 | WP_296789879.1 |
| cysteine peptidase family C39 domain-containing protein [Methanobrevibacter ruminantium] | *Methanobrevibacter ruminantium* | 133 | 133 | 94% | 7e-34 | 46.1 | 355 | WP_304094396.1 |
| cysteine peptidase family C39 domain-containing protein [Methanobrevibacter sp.] | *Methanobrevibacter sp.* | 131 | 131 | 99% | 1e-33 | 44.1 | 292 | WP_458405960.1 |
| MAG: hypothetical protein BZ136_07475 [Methanosphaera sp. rholeuAM74] | *Methanosphaera sp. rholeuAM74* | 129 | 129 | 96% | 3e-33 | 42.68 | 264 | RAP46439.1 |
| cysteine peptidase family C39 domain-containing protein [Methanobrevibacter sp.] | *Methanobrevibacter sp.* | 131 | 131 | 94% | 5e-33 | 45.45 | 355 | WP_407462773.1 |
| pseudomurein-binding repeat-containing protein [Methanobrevibacter smithii] | *Methanobrevibacter smithii* | 129 | 129 | 99% | 1e-32 | 44.44 | 294 | MFR3376057.1 |
| pseudomurein-binding repeat-containing protein [Methanobrevibacter intestini] | *Methanobrevibacter intestini* | 128 | 128 | 99% | 3e-32 | 44.44 | 307 | WP_427831960.1 |
| hypothetical protein [Methanobrevibacter smithii] | *Methanobrevibacter smithii* | 127 | 127 | 99% | 5e-32 | 44.44 | 308 | WP_252262434.1 |
| peptidoglycan-binding domain-containing protein [Methanosphaera sp. ISO3-F5] | *Methanosphaera sp. ISO3-F5* | 128 | 128 | 99% | 5e-32 | 41.36 | 342 | WP_323736286.1 |
| hypothetical protein [Methanobrevibacter smithii] | *Methanobrevibacter smithii* | 126 | 126 | 99% | 6e-32 | 43.83 | 271 | MDO5830902.1 |
| putative glycoside hydrolase [Methanobrevibacter sp.] | *Methanobrevibacter sp.* | 131 | 131 | 100% | 7e-32 | 42.41 | 606 | WP_405286286.1 |
| pseudomurein-binding repeat-containing protein [Methanobrevibacter smithii] | *Methanobrevibacter smithii* | 126 | 126 | 99% | 1e-31 | 43.83 | 295 | WP_400223183.1 |
| pseudomurein-binding repeat-containing protein [uncultured Methanobrevibacter sp.] | *uncultured Methanobrevibacter sp.* | 126 | 126 | 99% | 1e-31 | 43.21 | 307 | WP_295031596.1 |
| putative glycoside hydrolase [uncultured Methanobrevibacter sp.] | *uncultured Methanobrevibacter sp.* | 130 | 130 | 99% | 2e-31 | 42.07 | 562 | WP_296882086.1 |
| putative glycoside hydrolase [Methanobrevibacter sp.] | *Methanobrevibacter sp.* | 129 | 129 | 99% | 4e-31 | 45.06 | 596 | WP_458456230.1 |
| cysteine peptidase family C39 domain-containing protein [uncultured Methanobrevibacter sp.] | *uncultured Methanobrevibacter sp.* | 122 | 122 | 100% | 6e-31 | 41.77 | 225 | WP_299523216.1 |
| hypothetical protein [Methanobrevibacter sp.] | *Methanobrevibacter sp.* | 124 | 124 | 99% | 6e-31 | 43.21 | 302 | MBQ9026870.1 |
| hypothetical protein [Methanobrevibacter smithii] | *Methanobrevibacter smithii* | 121 | 121 | 99% | 1e-30 | 43.21 | 208 | WP_400258735.1 |
| hypothetical protein vir080_00065 [Caudoviricetes sp. vir080] | *Caudoviricetes sp. vir080* | 124 | 124 | 99% | 1e-30 | 43.83 | 308 | YP_013605401.1 |
| MAG TPA: hypothetical protein [Caudoviricetes sp.] | *Caudoviricetes sp.* | 122 | 122 | 99% | 3e-30 | 43.83 | 308 | DAY53184.1 |
| hypothetical protein [Methanobrevibacter smithii] | *Methanobrevibacter smithii* | 120 | 120 | 99% | 9e-30 | 43.21 | 271 | WP_400198677.1 |
| hypothetical protein [Methanobrevibacter smithii] | *Methanobrevibacter smithii* | 120 | 120 | 99% | 2e-29 | 42.59 | 308 | WP_400253395.1 |
| hypothetical protein [Methanobrevibacter sp.] | *Methanobrevibacter sp.* | 119 | 119 | 99% | 5e-29 | 39.51 | 308 | WP_303337379.1 |
| hypothetical protein HMCMC_EFILKL_02068 [uncultured Methanobrevibacter sp.] | *uncultured Methanobrevibacter sp.* | 114 | 114 | 67% | 6e-29 | 53.77 | 107 | CAQ7657118.1 |
| MAG TPA: Pseudomurein-binding repeat [Caudoviricetes sp.] | *Caudoviricetes sp.* | 118 | 118 | 100% | 1e-28 | 40.51 | 300 | DAU32704.1 |
| hypothetical protein [Methanobrevibacter sp.] | *Methanobrevibacter sp.* | 119 | 119 | 100% | 2e-28 | 41.14 | 327 | MBQ2654070.1 |
| hypothetical protein [Methanobrevibacter smithii] | *Methanobrevibacter smithii* | 117 | 117 | 99% | 2e-28 | 43.21 | 275 | WP_400231040.1 |
| hypothetical protein [Methanobrevibacter smithii] | *Methanobrevibacter smithii* | 118 | 118 | 99% | 2e-28 | 43.21 | 308 | WP_404808786.1 |
| hypothetical protein [Methanobrevibacter smithii] | *Methanobrevibacter smithii* | 117 | 117 | 99% | 2e-28 | 42.59 | 271 | MGN0140405.1 |
| MAG TPA: hypothetical protein [Caudoviricetes sp.] | *Caudoviricetes sp.* | 117 | 117 | 99% | 4e-28 | 42.59 | 308 | DAO11645.1 |
| hypothetical protein [Methanobrevibacter smithii] | *Methanobrevibacter smithii* | 116 | 116 | 99% | 6e-28 | 41.98 | 271 | MFR5821890.1 |
| peptidoglycan-binding domain-containing protein [Methanosphaera sp.] | *Methanosphaera sp.* | 117 | 117 | 99% | 1e-27 | 41.36 | 349 | WP_292474164.1 |
| hypothetical protein [Methanobrevibacter smithii] | *Methanobrevibacter smithii* | 115 | 115 | 99% | 2e-27 | 42.59 | 308 | HIQ8126120.1 |
| MAG TPA: Pseudomurein-binding repeat [Caudoviricetes sp.] | *Caudoviricetes sp.* | 115 | 115 | 100% | 3e-27 | 39.87 | 303 | DAU39951.1 |
| hypothetical protein [Methanobrevibacter smithii] | *Methanobrevibacter smithii* | 114 | 114 | 99% | 5e-27 | 41.98 | 308 | HJJ02104.1 |
| putative glycoside hydrolase [uncultured Methanobrevibacter sp.] | *uncultured Methanobrevibacter sp.* | 117 | 117 | 99% | 1e-26 | 40.37 | 588 | WP_296846991.1 |
| BACON domain-containing protein [Bacteroidales bacterium] | *Bacteroidales bacterium* | 117 | 117 | 99% | 1e-26 | 40.99 | 1323 | MBO4263708.1 |
| hypothetical protein [archaeon] | *archaeon* | 110 | 110 | 99% | 7e-26 | 36.42 | 243 | MCQ2821751.1 |
| C39 family peptidase [Methanobrevibacter ruminantium] | *Methanobrevibacter ruminantium* | 112 | 112 | 100% | 1e-25 | 36.78 | 363 | WP_304087592.1 |
| hypothetical protein [Methanobrevibacter sp.] | *Methanobrevibacter sp.* | 110 | 110 | 100% | 3e-25 | 41.14 | 320 | WP_475593720.1 |
| hypothetical protein [Methanosphaera sp.] | *Methanosphaera sp.* | 110 | 110 | 99% | 3e-25 | 39.13 | 342 | MBQ6219999.1 |
| hypothetical protein [Bacteroidales bacterium] | *Bacteroidales bacterium* | 110 | 110 | 99% | 2e-24 | 38.12 | 475 | MBQ9475631.1 |
| Ig-like domain repeat protein [Methanobrevibacter sp.] | *Methanobrevibacter sp.* | 110 | 110 | 99% | 2e-24 | 40.13 | 458 | MBQ2653059.1 |
| hypothetical protein [Methanobrevibacter ruminantium] | *Methanobrevibacter ruminantium* | 104 | 104 | 91% | 4e-24 | 38.67 | 186 | WP_304094132.1 |
| hypothetical protein [Methanobrevibacter sp.] | *Methanobrevibacter sp.* | 108 | 108 | 95% | 4e-24 | 41.33 | 378 | MBQ9024840.1 |
| hypothetical protein [Methanobrevibacter sp.] | *Methanobrevibacter sp.* | 105 | 105 | 100% | 7e-24 | 38.36 | 260 | MCL2115004.1 |
| BACON domain-containing protein [Bacteroidales bacterium] | *Bacteroidales bacterium* | 109 | 109 | 98% | 8e-24 | 38.99 | 948 | MBO4264344.1 |
| carboxypeptidase-like regulatory domain-containing protein [Methanobrevibacter sp.] | *Methanobrevibacter sp.* | 107 | 107 | 99% | 1e-23 | 37.58 | 370 | WP_476419353.1 |
| hypothetical protein [Methanobrevibacter sp.] | *Methanobrevibacter sp.* | 105 | 105 | 96% | 5e-23 | 38.41 | 374 | MBR0058971.1 |
| peptidoglycan-binding protein [Methanobrevibacter sp.] | *Methanobrevibacter sp.* | 103 | 103 | 99% | 1e-22 | 38.27 | 352 | MBO7210099.1 |
| Ig-like domain-containing protein [uncultured Methanobrevibacter sp.] | *uncultured Methanobrevibacter sp.* | 103 | 103 | 99% | 2e-22 | 40.13 | 368 | WP_296796147.1 |
| hypothetical protein [Turicibacter sp.] | *Turicibacter sp.* | 103 | 103 | 100% | 2e-22 | 37.43 | 361 | MBP3908438.1 |
| Ig-like domain-containing protein [uncultured Methanobrevibacter sp.] | *uncultured Methanobrevibacter sp.* | 103 | 103 | 99% | 2e-22 | 40.13 | 368 | WP_295612144.1 |
| cysteine peptidase family C39 domain-containing protein [Methanobrevibacter sp.] | *Methanobrevibacter sp.* | 102 | 102 | 99% | 3e-22 | 34.5 | 324 | WP_407415497.1 |
| hypothetical protein [Methanobrevibacter ruminantium] | *Methanobrevibacter ruminantium* | 102 | 102 | 91% | 5e-22 | 38.67 | 390 | WP_439778914.1 |
| BACON domain-containing protein [Bacteroidales bacterium] | *Bacteroidales bacterium* | 103 | 103 | 98% | 9e-22 | 37.11 | 833 | MBO4264033.1 |
| hypothetical protein [Candidatus Methanovirga meridionalis] | *Candidatus Methanovirga meridionalis* | 99.4 | 99.4 | 99% | 4e-21 | 32.91 | 331 | MDR3222612.1 |
| cysteine peptidase family C39 domain-containing protein [Methanobrevibacter sp.] | *Methanobrevibacter sp.* | 96.7 | 96.7 | 99% | 3e-20 | 38.85 | 296 | WP_475594130.1 |
| hypothetical protein [uncultured Methanobrevibacter sp.] | *uncultured Methanobrevibacter sp.* | 97.1 | 97.1 | 98% | 4e-20 | 35.98 | 325 | WP_296895142.1 |
| carboxypeptidase regulatory-like domain-containing protein [Methanobrevibacter sp.] | *Methanobrevibacter sp.* | 95.5 | 95.5 | 99% | 2e-19 | 36.94 | 376 | MBR0058553.1 |
| carboxypeptidase-like regulatory domain-containing protein [Methanobrevibacter sp.] | *Methanobrevibacter sp.* | 94.7 | 94.7 | 99% | 4e-19 | 39.49 | 371 | WP_407420665.1 |
| hypothetical protein [Methanobrevibacter sp.] | *Methanobrevibacter sp.* | 94 | 94 | 100% | 7e-19 | 37.34 | 377 | MBR0059608.1 |
| Ig-like domain repeat protein [Methanosphaera sp.] | *Methanosphaera sp.* | 93.2 | 93.2 | 100% | 4e-18 | 36.36 | 628 | MBQ6218471.1 |
| hypothetical protein [Methanobrevibacter millerae] | *Methanobrevibacter millerae* | 90.9 | 90.9 | 99% | 5e-18 | 38.22 | 314 | WP_303737712.1 |
| peptidoglycan-binding protein [Methanobacterium paludis] | *Methanobacterium paludis* | 89 | 89 | 98% | 1e-17 | 32.9 | 268 | MCE7699932.1 |
| hypothetical protein [Methanobrevibacter sp.] | *Methanobrevibacter sp.* | 90.5 | 90.5 | 100% | 3e-17 | 34.3 | 576 | MBR3140120.1 |
| hypothetical protein [Methanobrevibacter sp.] | *Methanobrevibacter sp.* | 88.2 | 88.2 | 99% | 1e-16 | 33.71 | 366 | MDO5818702.1 |
| hypothetical protein [Methanobacteriaceae archaeon] | *Methanobacteriaceae archaeon* | 87.8 | 87.8 | 100% | 1e-16 | 32.91 | 335 | MDD2644457.1 |
| hypothetical protein [Methanobrevibacter sp.] | *Methanobrevibacter sp.* | 87 | 87 | 100% | 1e-16 | 33.72 | 324 | MBR3140725.1 |
| hypothetical protein [uncultured Methanobrevibacter sp.] | *uncultured Methanobrevibacter sp.* | 86.7 | 86.7 | 99% | 2e-16 | 31.18 | 319 | WP_296886294.1 |
| BACON domain-containing protein [Bacteroidales bacterium] | *Bacteroidales bacterium* | 87.4 | 87.4 | 99% | 3e-16 | 31.25 | 952 | MBQ9476075.1 |
| pseudomurein-binding repeat-containing protein [Methanobrevibacter sp.] | *Methanobrevibacter sp.* | 85.9 | 85.9 | 99% | 6e-16 | 35.43 | 369 | WP_405288976.1 |
| putative glycoside hydrolase [uncultured Methanobrevibacter sp.] | *uncultured Methanobrevibacter sp.* | 86.7 | 86.7 | 99% | 7e-16 | 35.47 | 578 | WP_296852432.1 |
| hypothetical protein [Methanobrevibacter sp.] | *Methanobrevibacter sp.* | 82.4 | 82.4 | 99% | 1e-14 | 33.14 | 368 | MBQ6628063.1 |
| hypothetical protein [Methanobrevibacter ruminantium] | *Methanobrevibacter ruminantium* | 82 | 82 | 99% | 1e-14 | 31.43 | 369 | WP_304093665.1 |
| Ig-like domain repeat protein [Methanosphaera sp.] | *Methanosphaera sp.* | 82.4 | 82.4 | 96% | 2e-14 | 32.48 | 647 | MBQ6218472.1 |
| hypothetical protein HMCMC_ACHGAK_00044 [uncultured Methanobacteriaceae archaeon] | *uncultured Methanobacteriaceae archaeon* | 79.3 | 79.3 | 99% | 9e-14 | 32.48 | 301 | CAR3371688.1 |
| hypothetical protein [Paludibacteraceae bacterium] | *Paludibacteraceae bacterium* | 79.7 | 79.7 | 92% | 1e-13 | 32.45 | 356 | MBP5422977.1 |
| hypothetical protein [Candidatus Methanovirga basalitermitum] | *Candidatus Methanovirga basalitermitum* | 79.3 | 79.3 | 96% | 1e-13 | 29.22 | 327 | MDR2830897.1 |
| pseudomurein-binding repeat-containing protein [Methanobrevibacter sp. UBA417] | *Methanobrevibacter sp. UBA417* | 79 | 79 | 98% | 2e-13 | 31.61 | 338 | WP_413861801.1 |
| peptidoglycan-binding domain-containing protein [Methanobacterium sp.] | *Methanobacterium sp.* | 77.4 | 77.4 | 98% | 2e-13 | 32.92 | 252 | WP_424723327.1 |
| hypothetical protein [archaeon] | *archaeon* | 77.8 | 77.8 | 100% | 3e-13 | 29.71 | 282 | MCQ2964572.1 |
| cysteine peptidase family C39 domain-containing protein [Methanobrevibacter sp.] | *Methanobrevibacter sp.* | 79 | 79 | 99% | 4e-13 | 33.33 | 609 | WP_292812098.1 |
| hypothetical protein [Prevotella sp.] | *Prevotella sp.* | 76.6 | 76.6 | 100% | 4e-13 | 32.37 | 257 | MEE3417536.1 |
| hypothetical protein [Methanobrevibacter olleyae] | *Methanobrevibacter olleyae* | 77.8 | 77.8 | 99% | 6e-13 | 30.86 | 368 | WP_145884730.1 |
| hypothetical protein [Bacteroidales bacterium] | *Bacteroidales bacterium* | 75.5 | 75.5 | 99% | 5e-12 | 31.06 | 957 | MBQ9476078.1 |
| MULTISPECIES: hypothetical protein [Methanobacterium] | *Methanobacterium* | 73.9 | 73.9 | 99% | 1e-11 | 27.39 | 336 | WP_277896871.1 |
| MAG: hypothetical protein AMQ22_01331 [Candidatus Methanofastidiosum methylothiophilum] | *Candidatus Methanofastidiosum methylothiophilum* | 71.2 | 71.2 | 77% | 5e-11 | 30.58 | 254 | KYC51329.1 |
| hypothetical protein [uncultured Methanobrevibacter sp.] | *uncultured Methanobrevibacter sp.* | 69.3 | 69.3 | 77% | 6e-11 | 34.81 | 158 | WP_295722082.1 |
| hypothetical protein [Methanobrevibacter boviskoreani] | *Methanobrevibacter boviskoreani* | 68.2 | 68.2 | 99% | 1e-09 | 26.38 | 345 | WP_040682563.1 |
| Ig-like domain-containing protein [Methanobacterium sp. SMA-27] | *Methanobacterium sp. SMA-27* | 66.2 | 66.2 | 73% | 5e-09 | 35.29 | 343 | WP_231551454.1 |
| Ig-like domain-containing protein [Methanobacterium spitsbergense] | *Methanobacterium spitsbergense* | 66.2 | 66.2 | 73% | 7e-09 | 35.29 | 345 | WP_223790561.1 |
| pseudomurein-binding repeat-containing protein [Candidatus Hydrothermia bacterium] | *Candidatus Hydrothermia bacterium* | 65.5 | 65.5 | 98% | 1e-08 | 28.74 | 497 | HOP33408.1 |
| hypothetical protein [Candidatus Subteraquimicrobiales bacterium] | *Candidatus Subteraquimicrobiales bacterium* | 63.2 | 63.2 | 72% | 2e-08 | 27.83 | 202 | MDP3014149.1 |
| Ig-like domain repeat protein [Methanobrevibacter wolinii] | *Methanobrevibacter wolinii* | 62 | 62 | 99% | 2e-07 | 28.83 | 584 | WP_042708017.1 |
| C39 family peptidase [Methanobacterium lacus] | *Methanobacterium lacus* | 61.2 | 61.2 | 73% | 3e-07 | 33.61 | 284 | WP_013643849.1 |
| hypothetical protein [Methanobacterium sp.] | *Methanobacterium sp.* | 60.5 | 60.5 | 73% | 6e-07 | 34.45 | 282 | MTK63304.1 |
| endolysin [Glazvirus inraei] | *Glazvirus inraei* | 58.9 | 58.9 | 99% | 2e-06 | 31.76 | 259 | CAI4043391.1 |
| C39 family peptidase [Methanobacterium sp.] | *Methanobacterium sp.* | 57.4 | 57.4 | 76% | 3e-06 | 30.58 | 189 | HEY0196665.1 |
| C39 family peptidase [Methanobacterium sp. SMA-27] | *Methanobacterium sp. SMA-27* | 57.8 | 57.8 | 68% | 4e-06 | 32.43 | 271 | WP_156096015.1 |
| C39 family peptidase [Eubacteriales bacterium] | *Eubacteriales bacterium* | 56.6 | 56.6 | 38% | 1e-05 | 49.18 | 239 | MDD2484533.1 |
| C39 family peptidase [Methanobacterium sp.] | *Methanobacterium sp.* | 56.6 | 56.6 | 73% | 1e-05 | 30.51 | 296 | WP_424724863.1 |
| C39 family peptidase [Methanobacterium sp.] | *Methanobacterium sp.* | 56.6 | 56.6 | 73% | 1e-05 | 30.51 | 296 | WP_424715214.1 |
| C39 family peptidase [Synergistaceae bacterium] | *Synergistaceae bacterium* | 56.2 | 56.2 | 75% | 2e-05 | 31.34 | 420 | MGD9640708.1 |
| C39 family peptidase [Synergistaceae bacterium] | *Synergistaceae bacterium* | 56.2 | 56.2 | 75% | 2e-05 | 31.34 | 417 | MGE4487455.1 |
| C39 family peptidase [Brooklawnia cerclae] | *Brooklawnia cerclae* | 53.1 | 53.1 | 39% | 1e-04 | 44.44 | 178 | WP_167172123.1 |
| MAG: Peptidase C39 family protein [Methanobacterium sp. PtaU1.Bin097] | *Methanobacterium sp. PtaU1.Bin097* | 52 | 52 | 76% | 3e-04 | 28.93 | 188 | OPY20546.1 |
| C39 family peptidase [Methanobacteriaceae archaeon] | *Methanobacteriaceae archaeon* | 51.6 | 51.6 | 76% | 3e-04 | 28.93 | 188 | NYB27447.1 |
| C39 family peptidase [Thermoplasmatales archaeon] | *Thermoplasmatales archaeon* | 52 | 52 | 63% | 5e-04 | 30 | 260 | MBC7129280.1 |
| C39 family peptidase [Methanobacteriaceae archaeon] | *Methanobacteriaceae archaeon* | 52 | 52 | 89% | 5e-04 | 27.27 | 310 | HMK53233.1 |

**Table S4. Complete MOE docking energetic components for all compounds.**

| Compound | position | S (kcal/mol) | RMSD_refine (Å) | Key Interaction Residues |
| --- | --- | --- | --- | --- |
| **Sub 1** | 1 | -5.7599 | 2.6203 | G291 |
|  | 2 | -5.7538 | 2.7027 | G291 |
|  | 3 | -5.7156 | 1.5845 | Q194,C200 |
| **Sub 2** | 1 | -5.6892 | 0.8687 | K231,Q342 |
|  | 2 | -5.6193 | 3.6393 | S197,C200,K231 |
|  | 3 | -5.5881 | 2.6814 | C200,G291 |
| **Sub 3** | 1 | -6.2548 | 0.8526 | C200,T230,G291,H292 |
|  | 2 | -6.1607 | 2.3076 | Y290 |
|  | 3 | -5.9621 | 1.1624 | H283,G291 |
| **Sub 4** | 1 | -5.6896 | 1.4308 | Q194 |
|  | 2 | -5.6335 | 1.4168 | C200,T230 |
|  | 3 | -5.5563 | 1.7649 | C200,K231,G291 |
| **Sub 5*** | 1 | -5.5695 | 1.1478 | S197,Y321 |
|  | 2 | -5.5390 | 2.4481 | C200,Y290 |
|  | 3 | -5.5238 | 2.6039 | S197,C200,G291 |
| **Sub 6** | 1 | -5.5723 | 2.0345 | H283,H292 |
|  | 2 | -5.4894 | 2.0314 | H283,G291 |
|  | 3 | -5.4884 | 1.9981 | S197,C200 |
| **Sub 7** | 1 | -6.2094 | 1.5964 | T230,H283 |
|  | 2 | -6.1462 | 1.6358 | Q194,S197,C200,G291,H292 |
|  | 3 | -5.8852 | 1.0969 | S197 |
| **Sub 8** | 1 | -6.0285 | 1.5160 | no |
|  | 2 | -5.8085 | 1.4234 | K231,H283 |
|  | 3 | -5.5064 | 1.2385 | H283 |
| **Sub 9** | 1 | -5.8291 | 1.8457 | C200 |
|  | 2 | -5.7708 | 1.5303 | C200,K231,H283 |
|  | 3 | -5.7676 | 2.6513 | K231,T233 |
| **Sub 10** | 1 | -5.9278 | 1.5165 | G231 |
|  | 2 | -5.6790 | 1.2099 | S197,C200,Y290,G291 |
|  | 3 | -5.6251 | 1.3308 | S197 |

**Table S5. Kinetic parameters of R2S0-CD toward chromogenic substrates.**

| Substrate | Km (μM) | Vmax (μM/min) | kcat (min⁻¹) | kcat/Km (mM⁻¹·min⁻¹) |
| --- | --- | --- | --- | --- |
| Sub 5 (PNPG) | 372.9 ± 23.75 | 1.04 ± 0.03 | 0.208 ± 0.005 | (5.58 ± 0.38) × 10⁻^1^ |
| Sub 10 (ONPG) | 769.1 ± 79.29 | 0.99 ± 0.06 | 0.198 ± 0.011 | (2.57 ± 0.28) × 10⁻^1^ |

Values are presented as mean ± SD from three independent experiments.
